# Generation and Immune Regulation of CD4^+^CD25^−^Foxp3^+^ T Cells in Chronic Obstructive Pulmonary Disease

**DOI:** 10.3389/fimmu.2019.00220

**Published:** 2019-02-20

**Authors:** Jiang-Hua Wu, Mei Zhou, Yang Jin, Zhao-Ji Meng, Xian-Zhi Xiong, Sheng-Wen Sun, Shuai-Ying Miao, Hong-Li Han, Xiao-Nan Tao

**Affiliations:** Department of Respiratory and Critical Care Medicine, Union Hospital, Tongji Medical College, Huazhong University of Science and Technology, Wuhan, China

**Keywords:** COPD, CD4^+^CD25^−^Foxp3^+^ T cells, CD4^+^CD25^+^Foxp3^+^ T cells, Th17 cells, immune dysfunction

## Abstract

The imbalance of CD4^+^Foxp3^+^ T cell subsets is reportedly involved in abnormal inflammatory immune responses in patients with chronic obstructive pulmonary disease (COPD). However, the possible role of CD4^+^CD25^−^Foxp3^+^ T cells in immune regulation in COPD remains to be investigated. In the current study, distribution and phenotypic characteristics of CD4^+^CD25^−^Foxp3^+^ T cells from peripheral blood were determined by flow cytometry; the origin, immune function and ultimate fate of CD4^+^CD25^−^Foxp3^+^ T cells were further explored *in vitro*. It was observed that circulating CD4^+^CD25^−^Foxp3^+^ T cells were significantly increased in stable COPD patients (SCOPD) and resembled central memory or effector memory T cells. Compared with peripheral CD4^+^CD25^+^Foxp3^+^ T cells, peripheral CD4^+^CD25^−^Foxp3^+^ T cells showed a lower expression of Foxp3, CTLA-4, HELIOS, and TIGIT, but a higher expression of CD127 and KI-67, suggesting that CD4^+^CD25^−^Foxp3^+^ T cells lost the expression of Tregs-associated molecules following the reduction in CD25. Unexpectedly, our study found that transforming growth factor-β1 (TGFβ1) decreased CD25 expression and played a critical role in the generation of CD4^+^CD25^−^Foxp3^+^ T cells from CD4^+^CD25^+^Foxp3^+^ T cells. Phenotypic analysis further revealed that both inducible and peripheral CD4^+^CD25^−^Foxp3^+^ T cells exhibited the features of activated conventional T cells. Importantly, memory CD4^+^CD25^−^Foxp3^+^ T cells facilitated the proliferation and differentiation of naïve CD4^+^ T cells into Th17 cells in the presence of IL-1β, IL-6, IL-23, and TGFβ1. Finally, a fraction of CD4^+^CD25^−^Foxp3^+^ T cells, exhibiting instability and plasticity, were converted to Th17 cells when subjected to Th17 cell-polarizing condition. Taken together, we propose that TGFβ1 is responsible for the generation of CD4^+^CD25^−^Foxp3^+^ T cells, and these cells functionally exert an auxiliary effect on Th17 cells generation and might perpetuate chronic inflammation in COPD.

## Introduction

Chronic obstructive pulmonary disease (COPD) is characterized by persistent airflow limitation and progressive airway inflammation. Increasing evidence indicates that adaptive immune reactions are involved in the pathogenesis of COPD; in particular, inflammation mediated by T cells has been identified as a key component (1). Abnormalities in the number and function of CD4^+^ T cell subsets, including Th1 cells (2, 3), Th17 cells (4, 5), and regulatory T cells (Tregs) (6, 7), occur in the peripheral blood and lungs of patients with COPD, possibly influencing and perpetuating chronic inflammation.

COPD is not only characterized by mainly Th1-mediated type 1 inflammation (3, 8) but also by the imbalance between the anti-inflammatory subsets and the proinflammatory subsets of Tregs (7), which may play an important role in COPD progression. However, the molecular basis underlying the phenotypic and functional diversity of Tregs is incompletely understood. It is known that Foxp3 is transiently up-regulated in human CD4^+^CD25^−^ T cells *in vitro* after T cell receptor (TCR) stimulation, without acquisition of a suppressive function (9–12). Moreover, a population of Foxp3^+^ Tregs that constitutively express the T helper (Th) 17 lineage-specific transcription factor RAR-related orphan receptor (RORγt) has been identified in human, indicating that there is phenotypic and functional heterogeneity among Tregs (13). CD4^+^CD127^low^CD25^+^Foxp3^+^IL6R^hi^TIGIT^−^ T cells possess a potent *in vitro* suppressive capacity but display a distinct Th17 profile in the presence of IL-6-associated inflammation (14). An imbalance of circulating Th17 cells and Tregs results in immune dysfunction and the deterioration of pulmonary function in COPD (4, 15). Hence, it is urgent to elucidate the interplay between CD4^+^Foxp3^+^ T cells and Th17 cells in COPD patients.

Natural Tregs were initially recognized on the basis of their high expression of CD25(16). Thus, CD4^+^Foxp3^+^ T cells can be categorized into two subpopulations: CD4^+^CD25^+^Foxp3^+^ T cells and CD4^+^CD25^−^Foxp3^+^ T cells. Much attention has been given to CD4^+^CD25^+^Foxp3^+^ T cells for their role in the maintenance of immune homeostasis in COPD (6, 7, 17). However, the potential involvement of circulating CD4^+^CD25^−^Foxp3^+^ T cells in immune regulation in COPD is unknown. Although phenotypic and functional analysis of CD4^+^CD25^−^Foxp3^+^ T cells in autoimmune diseases such as systemic lupus erythematosus (SLE) and primary Sjögren‘s syndrome have been performed (18–23), there is still considerable controversy as to their function: Bonelli et al. proposed that increasing proportions of CD4^+^CD25^−^Foxp3^+^ T cells functionally resemble regulatory T cells in patients with SLE (22), whereas Yang et al. concluded that most CD4^+^CD25^−^Foxp3^+^ T cells are likely previously activated conventional T cells (23). Another recent study showed that CD4^+^CD25^low/−^Foxp3^+^ T cells represent a subpopulation of Tregs derived from CD4^+^CD25^high^Foxp3^+^ T cells in autoimmune diseases (18). Nonetheless, there has been almost no detailed study to date of the mechanism by which human CD4^+^CD25^−^Foxp3^+^ T cells differentiate and dynamically develop in chronic inflammatory diseases.

Our present study indicated that elevated percentages of peripheral CD4^+^CD25^−^Foxp3^+^ T cells were present in patients with stable COPD (SCOPD) and resembled central memory or effector memory T cells, and these cells were positively correlated with CD4^+^CD25^+^Foxp3^+^ T cells during exacerbation. Furthermore, we investigated the possible mechanism of origin, phenotypic characteristics, immune function and ultimate fate of CD4^+^CD25^−^Foxp3^+^ T cells in COPD patients.

## Materials and Methods

### Subjects

According to the diagnostic criteria for COPD from the GOLD 2016 guidelines, 28 patients with SCOPD, 24 patients with AECOPD, 18 asymptomatic smokers with normal lung function (healthy smokers, HS), and 22 asymptomatic healthy nonsmokers (healthy controls, HC) were enrolled (Table 1). All patients with SCOPD were initially diagnosed and had not received any systemic treatment including anticholinergics and glucocorticoids within 4 weeks prior the research. Patients with AECOPD were diagnosed at the initiation of exacerbated COPD symptoms, which required hospitalization, in the previous 72 h without any new therapeutic intervention. Subjects with a smoking history of ≥ 20 pack-years and normal lung function were defined as asymptomatic smokers. An ex-smoker was defined as an ever-smoker who had stopped smoking for at least 1 year. Subjects with malignant tumors, diabetes, coronary heart disease, and allergic and rheumatologic diseases were excluded. Peripheral blood samples were collected from all patients and volunteers. This study was conducted in accordance with the Declaration of Helsinki, and was approved by the Ethics Committee of Union Hospital, Tongji Medical College, Huazhong University of Science, and Technology (# 2013/S048). Written consent was obtained from every participant.

**Table 1:** Characteristics of all participants.

**Variables**	**HC**	**HS**	**SCOPD**	**AECOPD**
Subjects (No.)	22	18	28	24
Age (year)	57.3 ± 2.0	58.3 ± 1.9	58.7 ± 1.3	61.2 ± 1.2
Gender (Male/female)	16/6	14/4	24/4	20/4
Tobacco (pack-year)	–	44.7(20-76)	45.5(20-80)	46.5(24-78)
FEV1 (%predicted)	108.1 ± 2.3	105.6 ± 2.6	63.7 ± 4.4^*^#	57.4 ± 3.9^*^#
FEV1/FVC (%)	83.7 ± 1.4	84.6 ± 1.2	51.8 ± 2.4^*^#	49.3 ± 2.5^*^#
Corticosteroid use	0	0	0	14
**SMOKING STATUS**				
Current smokers	–	18	26	8
Ex-smokers	–	–	–	12

*The data are represented as the mean ± SEM or median (rang). FEV1, forced expiratory volume in 1 s; FVC, forced vital capacity*.

* P < 0.05 vs. HC subjects;

# *P < 0.05 vs. HS subjects*.

### Sample Collection and Processing

Peripheral blood samples were collected in heparin-treated tubes from each subject and used for plasma selection and peripheral blood mononuclear cell (PBMC) isolation from HC, HS and COPD patients within 1 h. The blood sample was immediately placed on ice and then centrifuged at 500 × g for 6 min. The cell-free supernatants of plasma were frozen at −80°C immediately after centrifugation for subsequent determination of cytokine concentrations. PBMCs were isolated from heparinized blood by Ficoll-Hypaque gradient centrifugation (Pharmacia, Uppsala, Sweden) to determine T cell subsets and purify subsets of CD4^+^ T cells.

### Cell Isolation

Naive CD4^+^ T cells from PBMC were isolated by negative selection with a naive CD4^+^ T cell isolation kit (Miltenyi Biotec, Bergisch-Gladbach, Germany). The purity of cells was ≥ 95%, as measured by flow cytometry.

CD4^+^CD25^−^Foxp3^+^ T cells were purified from TGFβ1-primed inducible CD4^+^CD25^+^Foxp3^+^ T cells with a CD25 MicroBead Kit (Miltenyi) for depletion of CD25^+^ cells, with an average purity of over 90%. For isolating inducible and peripheral CD4^+^CD25^−^CD127^+^Foxp3^+^ T cells and CD4^+^CD25^−^CD127^−^Foxp3^+^ T cells, CD4^+^CD25^−^CD45RO^+^CD127^+^ T cells, and CD4^+^CD25^−^CD45RO^+^CD127^−^ T cells were used to substitute for CD4^+^CD25^−^CD127^+^Foxp3^+^ T cells and CD4^+^CD25^−^CD127^−^Foxp3^+^ T cells, respectively. PBMC and TGFβ1-primed inducible CD4^+^CD25^+^Foxp3^+^ T cells were labeled with anti-CD4-FITC, anti-CD25-PE-Cy7, anti-CD45RO-PerCP-Cy5.5, and anti-CD127-PE. Afterwards, CD4^+^CD25^−^CD45RO^−^CD127^+^ T cells, CD4^+^CD25^−^CD45RO^+^CD127^+^ T cells, and CD4^+^CD25^−^CD45RO^+^CD127^−^ T cells were isolated by fluorescence-activated cell sorting (FACS), with an average purity >95%.

### Differentiation of CD4^+^CD25^–^Foxp3^+^ T Cells *in vitro*

Purified naive CD4^+^ T cells (5 × 10^5^) were cultured in 1 ml RPMI 1640 medium containing 10% fetal bovine serum (FBS) in 48-well plates and stimulated with plate-bound anti-CD3 (OKT3; 5 μg /ml; eBioscience, San Diego, USA) and soluble anti-CD28 mAb (5 μg/ml; eBioscience) in the presence of IL-2 (10 ng/ml; PeproTech, Rocky Hill, USA) for 7 days. Cultures were incubated at 37°C with 5% CO_2_. During culture, mix the cells with the pipette 10 times every 2 days. After 1 week of stimulation, harvest and rinse cells thoroughly with 60 ml PBS twice. Then, cells were re-cultivated in 1 ml RPMI 1640 medium containing 10% FBS in the presence of IL-2 (10 ng/ml) and TGFβ1 (10 ng/ml; PeproTech) for another 5–7 days.

To demonstrate that TGFβ1 was responsible for the generation of CD4^+^CD25^−^Foxp3^+^ T cells from CD4^+^CD25^+^Foxp3^+^ T cells, inducible CD4^+^CD25^+^Foxp3^+^ T cells were cultured in 1 ml of serum-free medium (X-VIVO 15TM, Lonza, Walkersville, MD, USA) in the presence of IL-2 (10 ng/ml) alone or in combination with different concentrations of TGFβ1 or anti-TGFβ1 (10 ng/ml; BioLegend, San Diego, USA) for 7 days. After stimulation, cells were analyzed for the expression of Foxp3 and CD25 by flow cytometry.

### Cytokine Detection

Concentrations of IL-1β, IL-6, IL-23, and TGFβ1 in plasma were measured by enzyme-linked immunosorbent assay (ELISA) kits according to the manufacturer's protocols (R&D Systems).

### Functional Assays

Cryopreserved autologous naïve CD4^+^ T cells (5 × 10^4^) were stained with carboxyfluorescein succinimididyl ester (CFSE, 5 μM; Invitrogen, Carlsbad, USA) and cultured in 96-well plates alone or co-cultured with unlabeled inducible CD4^+^CD25^−^Foxp3^+^ T cells at different ratios with stimulation of anti-CD3 (5 μg/ml) and soluble anti-CD28 mAbs (5 μg/ml) in serum-free medium supplemented with IL-2 (10 ng/ml). For transwell assays, CFSE-labeled naïve CD4^+^ T cells were seeded in the bottom chamber of a 24-well plate at a concentration of 5 × 10^5^ cells/well, and the unlabeled inducible CD4^+^CD25^−^Foxp3^+^ T cells were plated in the top chamber of the transwell (3-μm pore size) at 5 × 10^5^ cells/well. The top and bottom chambers of the transwell plates were coated with plate-bound anti-CD3 (5 μg/ml) and soluble anti-CD28 mAbs (5 μg/ml).

CFSE-labeled naïve CD4^+^ T cells (5 × 10^4^), activated with plate-bound anti-CD3 (5 μg/ml) and soluble anti-CD28 mAbs (5 μg/ml), were co-cultured in 96-well plates at a 1:1 ratio with FACS-sorted CD4^+^CD25^−^CD127^−^CD45RO^+^ T cells and CD4^+^CD25^−^CD127^+^CD45RO^+^ T cells in serum-free medium supplemented with IL-2 (10 ng/ml). Proliferation was measured by flow cytometry based on CFSE dilution after 4 days of culture. Regulatory activity was calculated by comparing the proliferation rate of naïve CD4^+^ T cells co-cultured with naïve CD4^+^ T cells with that of naïve CD4^+^ T cells co-cultured with indicated cells.

In certain experiments, CFSE-labeled autologous naïve CD4^+^ T cells (5 × 10^5^), activated with plate-bound anti-CD3 (5 μg/ml) and soluble anti-CD28 mAbs (5 μg/ml), were cultured alone or co-cultured at a 4:1 ratio with unlabeled inducible CD4^+^CD25^–^Foxp3^+^ T cells in 1 ml of serum-free medium containing human IL-2 (10 ng/ml) in the absence or presence of IL-1β (10 ng /ml) plus IL-6 (20 ng/ml) plus IL-23 (50 ng/ml) plus TGFβ1(10 ng/ml). After culturing for 5–7 days, cells were stimulated for 5 h with PMA (50 ng/ml; Sigma-Aldrich, St. Louis, USA) and ionomycin (1 μM; Sigma) in the presence of GolgiStop (BD Biosciences), and then harvested and intracellularly stained with anti-IL-17A mAbs; IL-17A^+^CFSE^+^ T cells were analyzed by flow cytometry. Additionally, the phenotype of naïve CD4^+^ T cells-derived progeny were subsequently analyzed for the expression of CCR7 and CD62L after stimulated under the above conditions.

### Flow Cytometry

The expression of surface markers and intracellular molecules of T cells were determined using flow cytometry. Cells were stained with fluorochrome-conjugated antibodies, which were purchased from BD Biosciences or eBioscience. List of Antibodies was presented in the Supplementary Table 1. Intracellular staining for IL-17A was performed after T cells were stimulated with PMA (50 ng /ml) and ionomycin (1 μM) in the presence of GolgiStop for 5 h. After fixation and permeabilization (eBioscience), intracellular proteins were labeled with the corresponding mAbs conjugated with fluorescent molecules, according to the manufacturer's instructions. Fixable viability stain 450 (BD Biosciences) was used to exclude dead cells. Flow cytometry was performed on a BD LSRFortessa X-20 and analyzed with FlowJo V10 software.

## The plasticity and Stability of Inducible CD4^+^Foxp3^+^ T Cells

Inducible CD4^+^CD25^+^Foxp3^+^ T cells (5 × 10^5^) were cultivated in 1 ml of serum-free medium containing human IL-2 (10 ng/ml) in the presence of the designated cytokines, either alone or in various combinations for 7 days. The exogenous cytokines used were TGFβ1 (10 ng/ml), IL-1β (10 ng/ml), IL-6 (20 ng/ml), and IL-23 (50 ng/ml). Portions of the cells were harvested to detect CD25, Foxp3, and RORC gene expression by qRT-PCR, as described below. The remaining cells were stimulated with PMA (50 ng/ml) and ionomycin (1 μM) in the presence of GolgiStop for 5 h, and the intracellular IL-17A were stained and then analyzed by flow cytometry as described above. In addition, inducible CD4^+^CD25^−^Foxp3^+^ T cells were sorted into CD4^+^CD25^−^CD127^−^Foxp3^+^ T cells and CD4^+^CD25^−^CD127^+^Foxp3^+^ T cells by FACS, and were cultivated in 1 ml of serum-free medium containing human IL-2 (10 ng/ml); their Foxp3 expression was detected by flow cytometry over time.

### Quantitative Real-Time PCR (qRT-PCR)

In brief, total RNA was extracted from cells using RNAiso plus (TaKaRa, Dalian, China) and reverse-transcribed into cDNA using a PrimeScriptTM RT Reagent Kit (TaKaRa) according to the manufacturer's protocol. Subsequently, PCR amplification was performed using a Step One Plus Real-Time PCR System (Applied Biosystems, Foster City, USA) using SYBR Premix Ex TaqTM (TaKaRa) and specific primers. The primer sequences were listed in the Supplementary Table 2. The expression level of GAPDH was used as an internal control. The qRT-PCR data were analyzed using StepOne software v2.3 (Applied Biosystems). The relative expression was quantified by the 2-ΔΔCt method to compare the differences among groups.

### Statistics

Data are expressed as the mean ± SEM (unless indicated in the figure legends). Comparisons of the data between different groups were evaluated by either two-tailed unpaired *t*-test or one-way analysis of variance (ANOVA) followed by Bonferroni‘s multiple comparisons test. Comparison of immune phenotypes among CD4^+^ T cell subsets from the same individual was performed using a paired 2-tailed Student's *t*-test. Correlations between variables were determined using the Spearman rank test. All statistical data were analyzed using GraphPad Prism 6 software (GraphPad Software, La Jolla, California), and *P* < 0.05 was considered statistically significant.

## Results

### Frequency of Peripheral CD4^+^CD25^−^Foxp3^+^ T Cells Is Increased in SCOPD Patients

**Figure 1:**
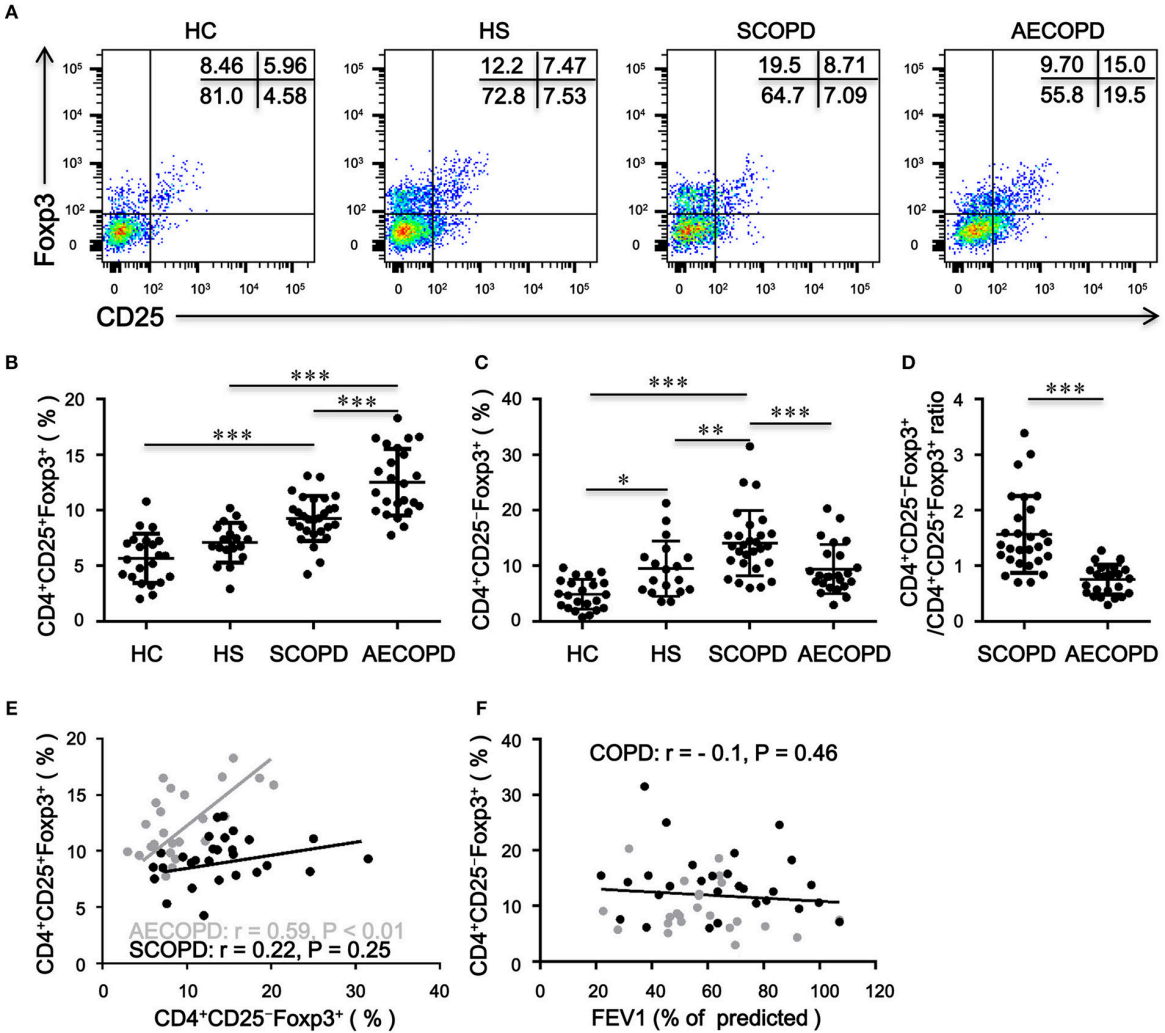
Frequency of circulating CD4^+^CD25^–^Foxp3^+^ T cells and CD4^+^CD25^+^Foxp3^+^ T cells in patients with chronic obstructive pulmonary disease (COPD). **(A)** Representative flow cytometric dot plots of CD4^+^CD25^–^Foxp3^+^ T cells and CD4^+^CD25^+^Foxp3^+^ T cells within CD4^+^ T cells in healthy controls (HC), healthy smokers (HS), stable COPD patients (SCOPD), and patients with acute exacerbation of COPD (AECOPD). Comparisons of CD4^+^CD25^−^Foxp3^+^ T cell **(B)** and CD4^+^CD25^+^Foxp3^+^ T cell **(C)** percentages in peripheral blood from HC (*n* = 22), HS (*n* = 18), SCOPD (*n* = 28), and AECOPD (*n* = 24) patients. Horizontal bars indicate means ± SD. **(D)** Comparisons of ratios of CD4^+^CD25^−^Foxp3^+^ T cells/CD4^+^CD25^+^Foxp3^+^ T cells in peripheral blood from SCOPD (*n* = 28) and AECOPD (*n* = 24). **(E)** and **(F)** Correlation of the percentages of CD4^+^CD25^−^Foxp3^+^ T cells with the percentages of CD4^+^CD25^+^Foxp3^+^ T cells and FEV1 (% of predicted) in COPD patients; each symbol represents an individual SCOPD patient (black dots) or AECOPD patients (gray dots). Correlations were determined by Spearman‘s rank correlation coefficients. The comparisons were determined by one-way ANOVA followed by Bonferroni‘s multiple comparison test **(B,C)** or two-tailed unpaired *t*-test **(D)**, ^*^P < 0.05, ^**^P < 0.01, ^***^P < 0.001.

Patients with AECOPD had significantly elevated percentages of CD4^+^CD25^+^Foxp3^+^ T cells compared with HC, HS and patients with SCOPD (Figures 1A,B). Inversely, the frequency of CD4^+^CD25^−^Foxp3^+^ T cells was markedly increased in patients with SCOPD compared to HC and patients with AECOPD (Figures 1A,C). Interestingly, the ratio of CD4^+^CD25^−^Foxp3^+^ T cells/CD4^+^CD25^+^Foxp3^+^ T cells was significantly higher in SCOPD than in AECOPD patients (Figure 1D), and single regression analysis suggested a positive correlation for the percentage of CD4^+^CD25^−^Foxp3^+^ T cells with CD4^+^CD25^+^Foxp3^+^ T cells in AECOPD (*r* = 0.54, *P* < 0.001; Figure 1E) but not in SCOPD, suggesting a potential mutual transformation between these cells in the different phases of the disease. However, the frequency of CD4^+^CD25^−^Foxp3^+^ T cells showed no correlation with lung function in COPD patients (*r* = -0.10, *P* = 0.46; Figure 1F). Taken together, these data indicate that the frequency of CD4^+^CD25^−^Foxp3^+^ T cells does not correlate with lung function but might reflect disease activity in COPD patients.

### TGFβ1 Reduced the Expression of CD25 and Generated CD4^+^CD25^−^Foxp3^+^ T Cells From CD4^+^CD25^+^Foxp3^+^ T Cells *in vitro*

**Figure 2:**
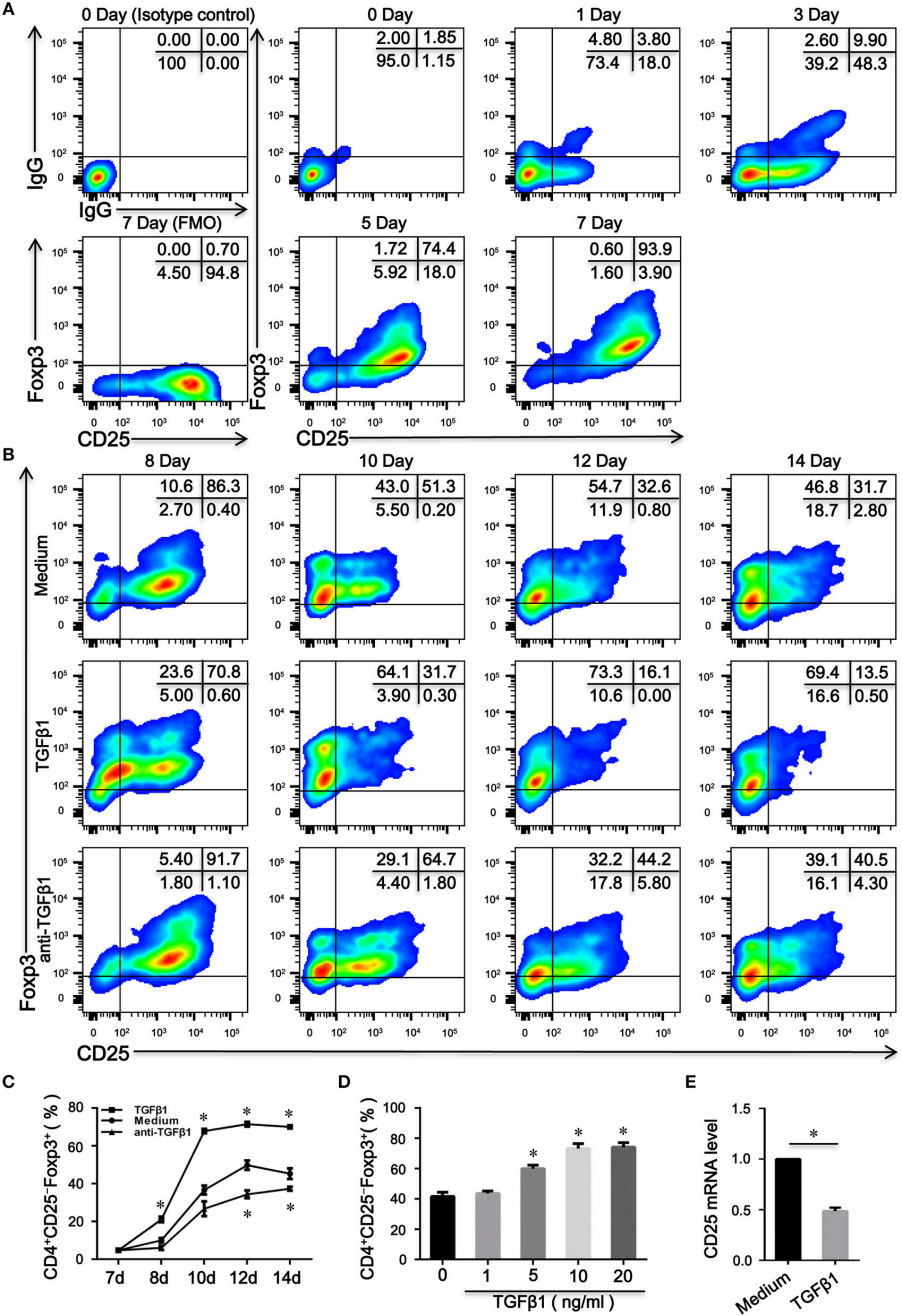
TGFβ1 is responsible for the generation of CD4^+^CD25^–^Foxp3^+^ T cells from CD4^+^CD25^+^Foxp3^+^ T cells. **(A)** Representative dot plots detecting the expression of CD25 and Foxp3 in total cells after naive CD4^+^ T cells were stimulated with plate-bound anti-CD3 and soluble anti-CD28 mAb in the presence of IL-2 for 7 days. **(B)** Representative dot plots detecting the expression of CD25 and Foxp3 in total cells after inducible CD4^+^CD25^+^Foxp3^+^ T cells were cultured in the presence of IL-2 alone or combined with TGFβ1 or with anti-TGFβ1 for another 7 days. **(C)** Line graph of changes over time in the percentages of CD4^+^CD25^−^Foxp3^+^ T cells after inducible CD4^+^CD25^+^Foxp3^+^ T cells were cultured in the above condition. Data are presented as mean ± SEM of 4 independent experiments; ^*^P < 0.05 compared with medium control at the same time point. **(D)** Summary graph indicating the percentage of CD4^+^CD25^−^Foxp3^+^ T cells in each group after inducible CD4^+^CD25^+^Foxp3^+^ T cells were stimulated by different concentrations of TGFβ1. Data are presented as mean ± SEM of 4 independent experiments; ^*^P < 0.05 compared with medium control. **(E)** Summary graph displaying relative mRNA expression levels of CD25 in inducible CD4^+^CD25^+^Foxp3^+^ T cells after stimulated with or without TGFβ1. Data are presented as mean ± SEM of 5 independent experiments; ^*^P < 0.05 compared with medium control. The comparisons were determined by one-way ANOVA followed by Bonferroni‘s multiple comparison test **(C,D)** or two-tailed unpaired *t*-test **(E)**.

CD4^+^CD25^low/−^Foxp3^+^ T cells are reportedly derived from CD4^+^CD25^high^Foxp3^+^ T cells in SLE patients (18). However, the mechanism by which human CD4^+^CD25^−^Foxp3^+^ T cells differentiate remains to be elucidated. Having established that TCR stimulation alone can induce CD25 and Foxp3 expression in human CD4^+^CD25^−^ T cells (9, 10), we also observed that CD4^+^CD25^+^Foxp3^+^ T cells (>85%) were induced when naïve CD4^+^ T cells were activated through TCR stimulation independent of exogenous TGFβ1 (Figure 2A); moreover, TGFβ1 stimulation did not influence CD25 expression on naïve CD4^+^ T cells (Data is not shown). Then, inducible CD4^+^CD25^+^Foxp3^+^ T cells were harvested and re-cultivated in the presence of IL-2 alone or combined with TGFβ1 or with anti-TGFβ1 for another 7 days. As shown in Figures 2B–D, unexpectedly, the expression of CD25 on CD4^+^CD25^+^Foxp3^+^ T cells was decreased by TGFβ1 in a dose-dependent and time-dependent manner; these effects were delayed by a neutralizing anti-TGFβ1 monoclonal antibody (mAb); moreover, inducible CD4^+^CD25^+^Foxp3^+^ T cells lost CD25 expression when cultured in serum-free medium in the presence of IL-2, suggesting that these cells could secret TGFβ1. We utilized qRT-PCR to further demonstrate that CD25 mRNA expression level in inducible CD4^+^CD25^+^Foxp3^+^ T cells was decreased by TGFβ1 (Figure 2E). Together, we conclude that CD4^+^CD25^−^Foxp3^+^ T cells originate from CD4^+^CD25^+^Foxp3^+^ T cells via the influence of TGFβ1.

### Phenotypic Characterization of CD4^+^CD25^−^Foxp3^+^ T Cells

**Figure 3:**
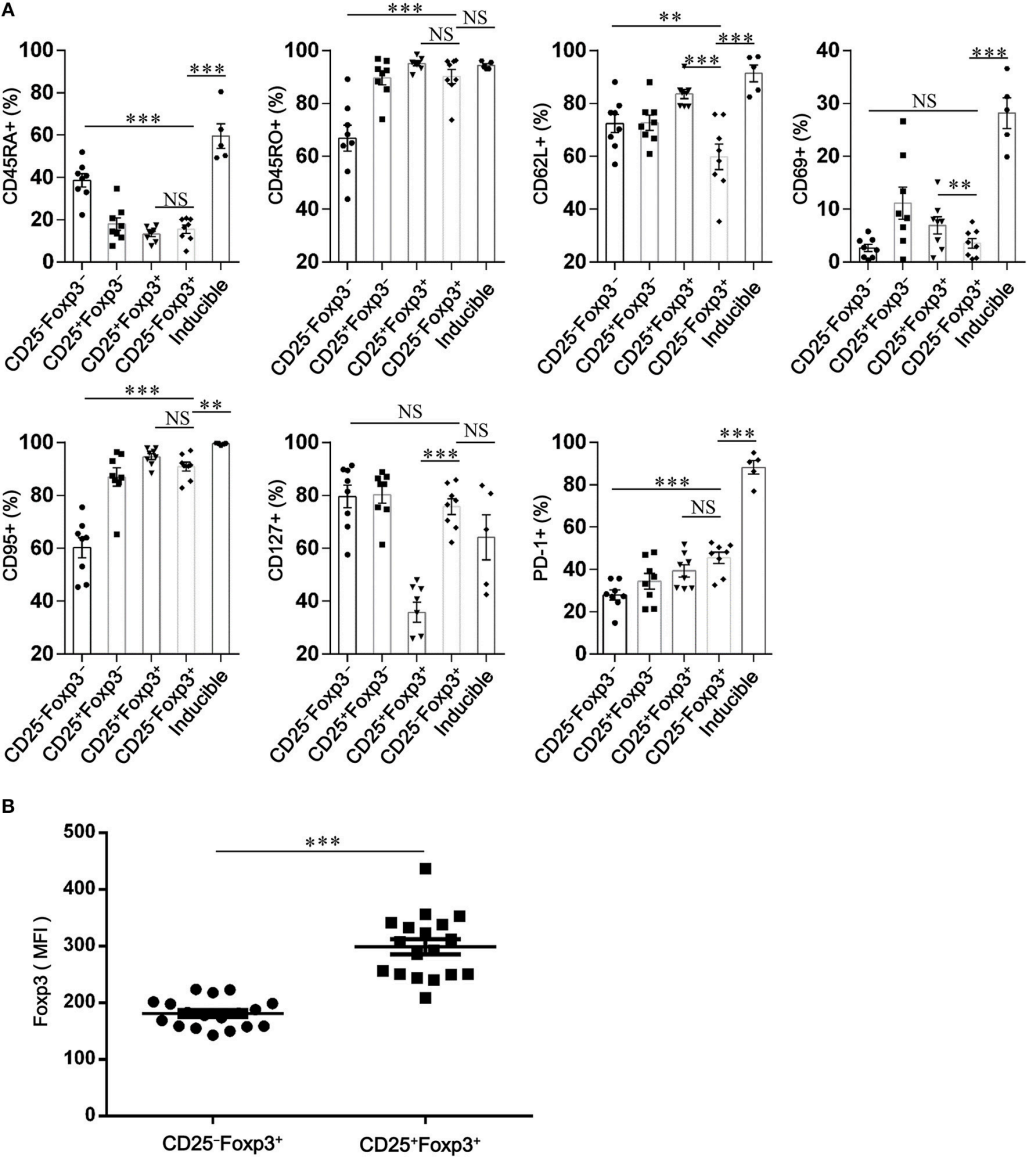
CD4^+^CD25^−^Foxp3^+^ T cells resemble activated conventional T cells in patients with chronic obstructive pulmonary disease (COPD). **(A)** Scatter plots depict the distribution of CD45RA, CD45RO, CD62L, CD69, CD95, CD127, and PD-1 amongst: peripheral CD4^+^ T cells subsets (CD4^+^CD25^−^Foxp3^−^ T cells, CD4^+^CD25^+^Foxp3^−^ T cells, CD4^+^CD25^+^Foxp3^+^ T cells and CD4^+^ CD25^−^Foxp3^+^ T cells) from SCOPD patients (*n* = 8) and inducible CD4^+^CD25^−^Foxp3^+^ T cells (Inducible, *n* = 5). Horizontal bars indicate means ± SEM. The comparisons among peripheral CD4^+^ T cells subsets were made using two-tailed paired *t*-test; the comparisons between peripheral CD4^+^ T cells subsets and inducible CD4^+^CD25^−^Foxp3^+^ T cells were made using two-tailed unpaired *t*-test, ^*^P < 0.05, ^**^P < 0.01, ^***^P < 0.001. **(B)** Expression of Foxp3 in peripheral CD4^+^CD25^+^Foxp3^+^ T cells and CD4^+^CD25^−^Foxp3^+^ T cells from COPD patients (*n* = 18) was measured by mean fluorescence intensity (MFI). Horizontal bars indicate means ± SEM. The comparisons were determined by two-tailed paired *t*-test, ^***^P < 0.001 compared with CD4^+^CD25^+^Foxp3^+^ T cells.

To identify the phenotypic characteristics of peripheral CD4^+^CD25^−^Foxp3^+^ T cells in patients with SCOPD, we analyzed the expression profiles of CD45RA, CD45RO, CD62L, CD69, CD95, and programmed cell death-1 (PD-1). For most of these cells, high levels of CD45RO (90.2 ± 2.7%) and CD95 (90.8 ± 1.7%), but not of CD45RA (15.5 ± 1.9%), CD69 (3.5 ± 0.9%), and CD62L (59.8 ± 4.8%), were observed in patients with SCOPD, indicating that these cells were central memory or effector memory T cells (Figure 3A; Figure S1). Furthermore, compared to CD4^+^CD25^−^Foxp3^−^ T cells, CD4^+^CD25^−^Foxp3^+^ T cells had a higher expression of PD-1, which was defined by a characteristic of T cell activation or dysfunction (Figure 3A; Figure S1).

**Figure 4:**
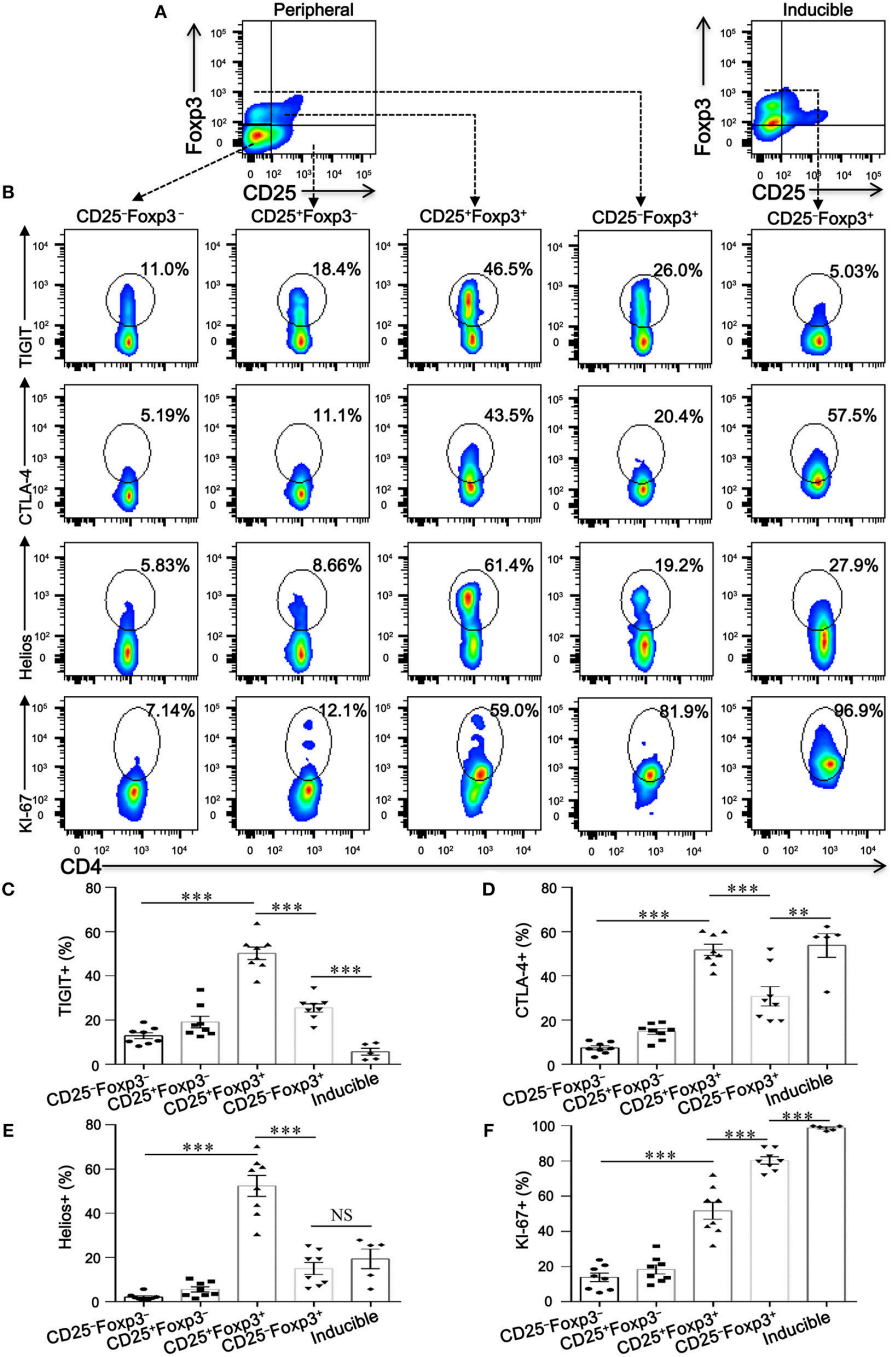
CD4^+^CD25^−^Foxp3^+^ T cells lose classical markers relevant to Tregs. **(A)** Gating strategy for CD4^+^ T cell subsets based on CD25 and Foxp3 expression. **(B)** Representative dot plots for the detection of the expression of the conventional Treg markers TIGIT, CTLA-4, Helios, and KI-67 amongst: peripheral CD4^+^ T cells subsets (CD4^+^CD25^−^Foxp3^−^ T cells, CD4^+^CD25^+^Foxp3^−^ T cells, CD4^+^CD25^+^Foxp3^+^ T cells and CD4^+^CD25^−^Foxp3^+^ T cells) from SCOPD patients (*n* = 8) and inducible CD4^+^CD25^−^Foxp3^+^ T cells (Inducible, *n* = 5). **(C–F)** Scatter plots depict the distribution of TIGIT, CTLA-4, Helios, and KI-67 in the five assessed CD4^**+**^ T cell subsets. Horizontal bars indicate means ± SEM. The comparisons among peripheral CD4^+^ T cells subsets from the same individual were made using two-tailed paired *t*-test; the comparisons between peripheral CD4^+^ T cells subsets and inducible CD4^+^CD25^−^Foxp3^+^ T cells were made using two-tailed unpaired *t*-test, ^**^P < 0.01, ^***^P < 0.001.

To investigate whether CD4^+^CD25^−^Foxp3^+^ T cells share phenotypic features resembling conventional CD4^+^CD25^+^Foxp3^+^ T cells in COPD patients, we analyzed the expression profiles of some classical markers relevant to Tregs, such as CD127, CTLA-4, HELIOS, TIGIT, and KI-67. Compared with CD4^+^CD25^+^Foxp3^+^ T cells, CD4^+^CD25^−^Foxp3^+^ T cells showed a significantly lower expression of Foxp3, CTLA-4, HELIOS, and TIGIT, but a higher expression of KI-67 and CD127 (Figures 3, 4; Figure S1), suggesting that these cells lost the expression of Tregs-associated molecules following the reduction in CD25.

We next addressed whether the phenotypic characteristics of inducible CD4^+^CD25^−^Foxp3^+^ T cells were consistent with those found in peripheral blood. Overall, inducible CD4^+^CD25^−^Foxp3^+^ T cells expressed higher levels of CD45RA, CD62L, CD69, CD95, CTLA-4, KI-67, and PD-1 compared with peripheral CD4^+^CD25^−^Foxp3^+^ T cells, and no difference in CD45RO, HELIOS, and CD127 expression was observed between the two cell populations; meanwhile, both peripheral and inducible CD4^+^CD25^−^Foxp3^+^ T cells expressed low levels of HELIOS and TIGIT (Figures 3, 4; Figure S1). Although the expression pattern, especially for several activation-associated marker molecules, is not identical between inducible and peripheral CD4^+^CD25^−^Foxp3^+^ T cells, most of these cells exhibit phenotypic features of activated conventional T cells. In conclusion, our findings highlight a hitherto unknown pathway for the differentiation of CD4^+^CD25^−^Foxp3^+^ T cells from CD4^+^CD25^+^Foxp3^+^ T cells via the influence of TGFβ1.

### Memory CD4^+^CD25^−^Foxp3^+^ T Cells Functionally Facilitate Naïve CD4^+^ T Cells Proliferation *in vitro*

**Figure 5:**
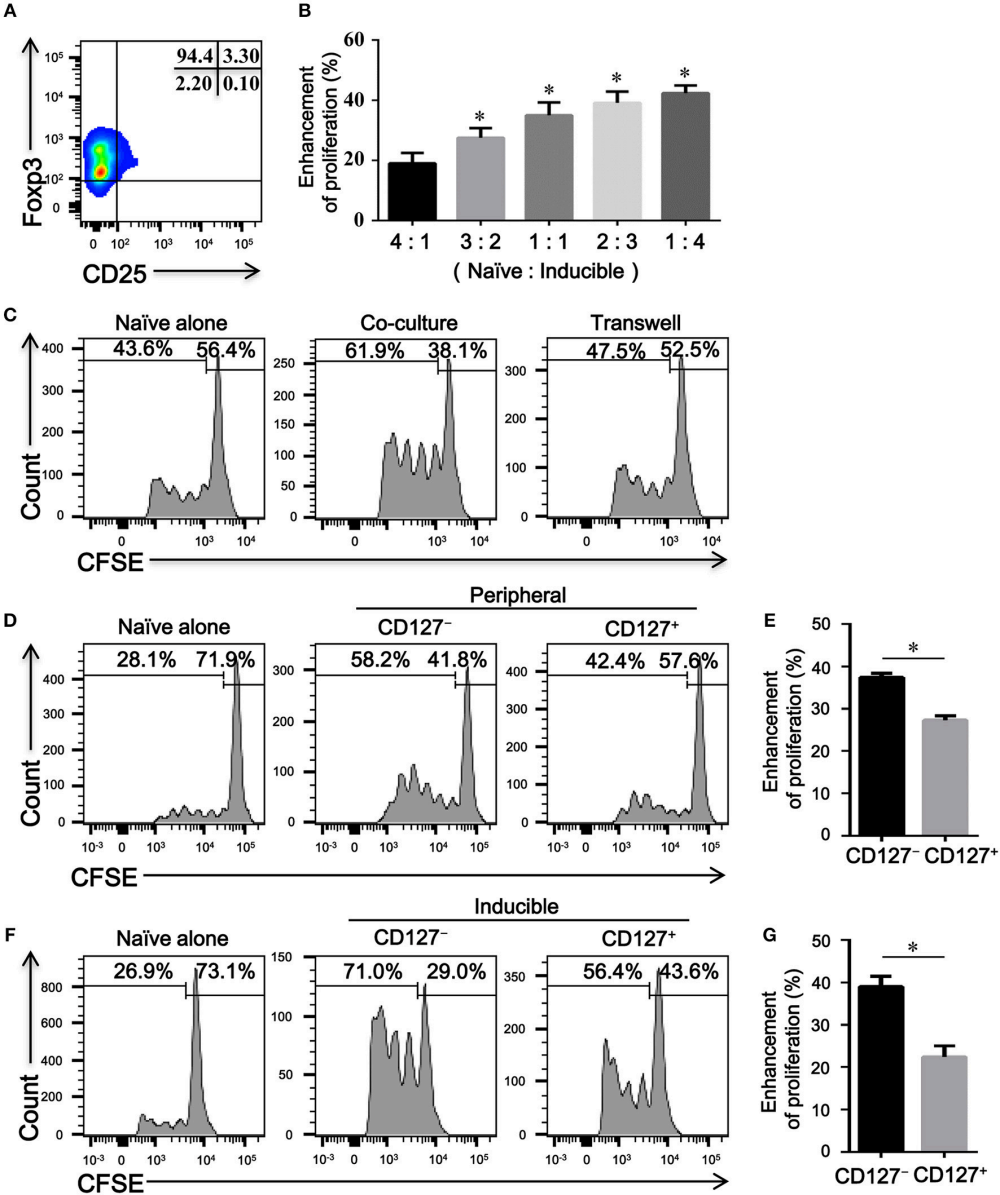
CD4^+^CD25–Foxp3^+^ T cells enhance the proliferation of naïve CD4^+^ T cells *in vitro*. **(A)** A representative dot plot showing that a high purity of CD4^+^CD25^−^Foxp3^+^ T cells could be obtained *in vitro*. **(B)** CFSE-labeled autologous naïve CD4^+^ T cells (Naive) isolated from HC, activated with plate-bound anti-CD3/CD28 mAb, were co-cultured with indicated ratio of inducible CD4^+^CD25^−^ Foxp3^+^ T cells (Inducible) for 4 days. The proliferation was evaluated by flow cytometry after stimulation. Summary graph showing the percentages of the enhancement of proliferation by different ratios of inducible CD4^+^CD25^−^Foxp3^+^ T cells in each group, indicated by progressive numbers. The results are reported as means ± SEM from 5 independent experiments; ^*^P < 0.05 compared with 4:1 ratio control. **(C)** Transwell experiments showing that the enhanced proliferation by inducible CD4^+^CD25–Foxp3^+^ T cells was cell-contact dependent. Representative histograms are from one of 3 independent experiments. **(D)** CFSE-labeled autologous naïve CD4^+^ T cells (Naïve), activated with plate-bound anti-CD3/CD28 mAb, were co-cultured at a ratio of 1:1 with FACS-isolated peripheral CD4^+^CD25^−^CD45RO^+^CD127^+^ T cells (CD127^+^) or CD4^+^CD25^−^CD45RO^+^CD127^−^ T cells (CD127^−^) for 4 days. Representative histograms showing the proliferation of CFSE-labeled naïve T cells. **(F)** CFSE-labeled autologous naïve CD4^+^ T cells (Naïve), activated with plate-bound anti-CD3/CD28 mAb, were co-cultured at a ratio of 1:1 with FACS-isolated inducible CD4^+^CD25^−^CD45RO^+^CD127^+^ T cells (CD127^+^) or CD4^+^CD25^–^CD45RO^+^CD127^–^ T cells (CD127^–^) for 4 days. Representative histograms showing the proliferation of CFSE-labeled naïve T cells. Graphs **(E,G)** represented summary data **(D,F)**, respectively. Summary graph showing the percentages of the enhancement of proliferation by CD4^+^CD25^−^CD45RO^+^CD127^+^ T cells (CD127^+^) and CD4^+^CD25^−^CD45RO^+^CD127^−^ T cells (CD127^−^) in each group, indicated by progressive numbers. The results are reported as means ± SEM from 5 independent experiments; ^*^P < 0.05 compared with CD127^+^. The comparisons were determined by one-way ANOVA followed by Bonferroni‘s multiple comparison test **(B)** or two-tailed unpaired *t*-test **(E,G)**.

To determine the immune capacity of inducible CD4^+^CD25^−^Foxp3^+^ T cells *in vitro*, we next purified these cells from TGFβ1-primed CD4^+^CD25^+^Foxp3^+^ T cells with a purity of over > 90% (Figure 5A). As shown in Figures 5B,C, CD4^+^CD25^−^Foxp3^+^ T cells enhanced the proliferation of naïve CD4^+^ T cells in cell-dose, contact, and activation dependent manner. Since CD127 expression is inversely correlated with the suppressive function of Tregs (24), we further evaluated the regulatory activity of CD4^+^CD25^−^CD127^+^Foxp3^+^ T cells and CD4^+^CD25^−^CD127^−^Foxp3^+^ T cells *in vitro*. As peripheral CD4^+^CD25^−^Foxp3^+^ T cells expressed high levels of CD45RO in COPD patients (Figure 3A), CD4^+^CD25^−^CD45RO^+^CD127^+^ T cells and CD4^+^CD25^−^CD45RO^+^CD127^−^ T cells were used as substitutes for CD4^+^CD25^−^CD127^+^Foxp3^+^ T cells and CD4^+^CD25^−^CD127^−^ Foxp3^+^ T cells, respectively; 27.4 ± 2.8% of peripheral CD4^+^CD25^−^CD45RO^+^CD127^−^ T cells, in comparison to 15.5 ± 2.5 % of peripheral CD4^+^CD25^−^CD45RO^+^CD127^+^ T cells, expressed Foxp3 (Figure S2). As shown in Figures 4D–G, both peripheral and inducible CD4^+^CD25^−^CD45RO^+^CD127^+^ T cells and CD4^+^CD25^−^CD45RO^+^CD127^−^ T cells were equally capable of augmenting the proliferation of naïve CD4^+^ T cells; unexpectedly, CD4^+^CD25^−^CD45RO^+^CD127^−^ T cells exhibited much greater enhancement of naïve CD4^+^ T cells proliferation than did CD4^+^CD25^−^CD45RO^+^CD127^+^ T cells *in vitro*.

Whether the progressive enhancement in the proliferation of naïve T cell is exerted by CD4^+^CD25^–^Foxp3^+^ T cells in COPD patients has not been previously evaluated. We next compared the effects of peripheral CD4^+^CD25^−^CD45RO^+^CD127^+^ T cells and CD4^+^CD25^−^CD45RO^+^CD127^−^ T cells from HC and COPD patients on the proliferation of the same naïve CD4^+^ T cell. According to this heterologous co-culture assay, both CD4^+^CD25^−^CD45RO^+^CD127^+^ T cells and CD4^+^CD25^−^CD45RO^+^CD127^−^ T cells from COPD patients exhibited the enhancement capacity equal to those cells from HC subjects (Figure S3).

### Increased Proportions of Th17 Cells may be Partially due to CD4^+^CD25^−^Foxp3^+^ T Cells in COPD Patients

**Figure 6:**
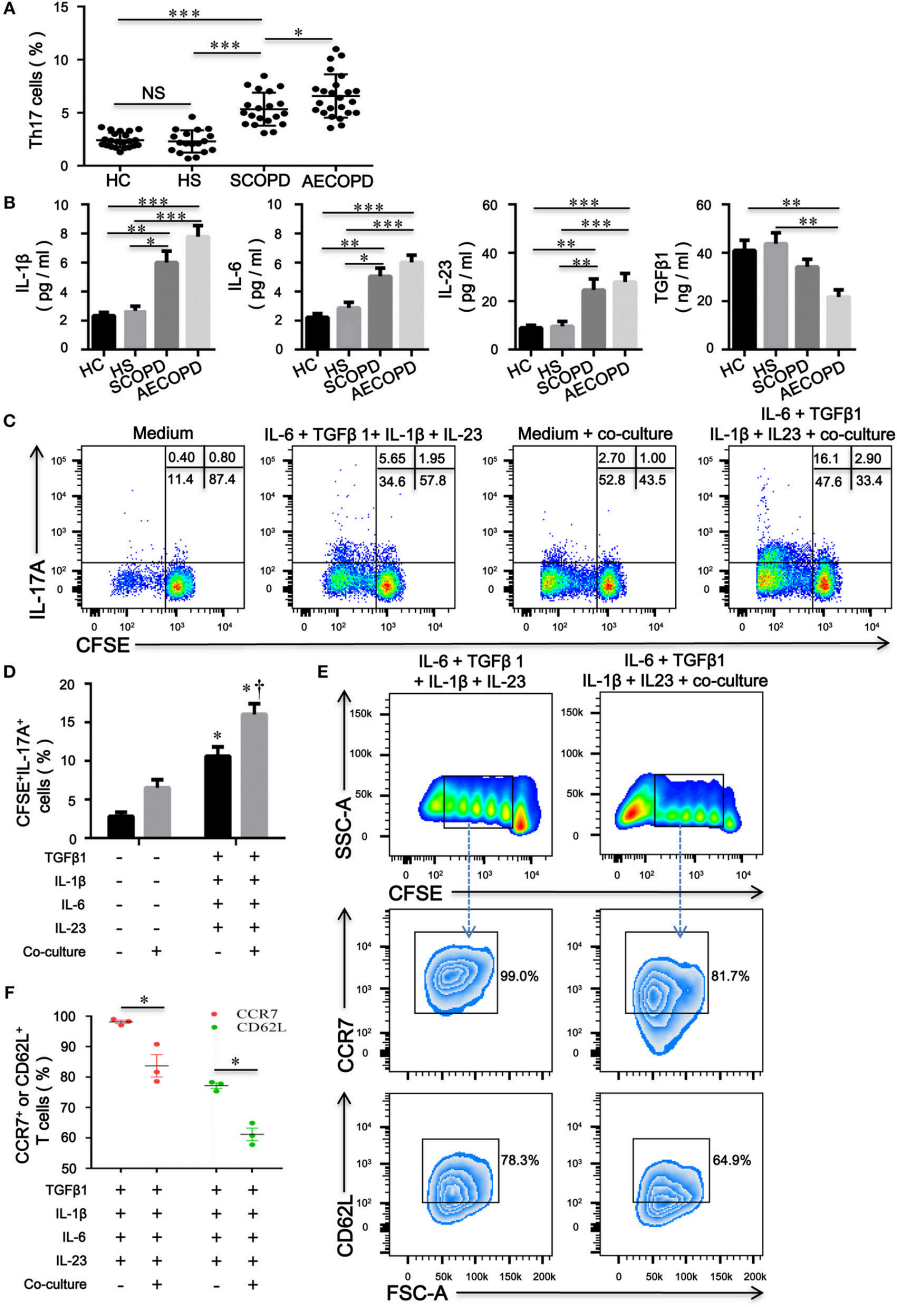
CD4^+^CD25^–^Foxp3^+^ T cells facilitate the differentiation of naïve CD4^+^ T cells into Th17 cells in patients with chronic obstructive pulmonary disease (COPD). **(A)** Th17 cells within CD4^+^ T cells were identified on the basis of their expression of CD3 and not of CD8; the comparison of Th17 cell percentages in peripheral blood from HC (*n* = 22), HS (*n* = 18), SCOPD (*n* = 20), and AECOPD (*n* = 24) patients was performed. Horizontal bars indicate means ± SD; ^*^P < 0.05, ^**^P < 0.01, ^***^P < 0.001. **(B)** Comparisons of concentrations of IL-1β, IL-6, IL-23, and TGFβ1 in peripheral blood from HC (*n* = 22), HS (*n* = 18), SCOPD (*n* = 20), and AECOPD (*n* = 24) patients; the results are reported as means ± SEM, ^*^P < 0.05, ^**^P < 0.01, ^***^P < 0.001. **(C)** Representative dot plots showing the gating strategy for the detection of CFSE^+^IL-17A^+^ T cells after CFSE**-**labeled naïve CD4^+^ T cells from COPD patients, activated with plate-bound anti-CD3/CD28 mAbs, were cultured alone or co-cultured at a 4:1 ratio with unlabeled inducible CD4^+^CD25^−^Foxp3^+^ T cells under the designated conditions for 5 days. **(D)** Comparisons of the percentages of CFSE^+^IL-17A^+^ T cells in each group. The results are reported as means ± SEM from 5 independent experiments; ^*^P < 0.05 compared with medium control; ^†^*P* < 0.05 compared with IL-1β plus IL-6 plus IL-23 plus TGFβ1 control. **(E)** Representative dot plots showing the gating strategy for the detection of phenotype in naïve CD4^+^ T cells-derived progeny 5 days following the activation alone or in the presence of inducible CD4^+^CD25^−^Foxp3^+^ T cells at a ratio of 4:1. Naïve CD4^+^ T cells were labeled with CFSE and stimulated with plate-bound anti-CD3/CD28 mAbs under the designated conditions. Naïve CD4^+^ T cells-derived progeny were subsequently analyzed for the expression of CCR7 and CD62L. **(F)** Comparisons of the percentages of CCR7^+^ or CD62L^+^ T cells in naïve-derived progeny in each group. The results are reported as means ± SEM from 3 independent experiments; ^*^P < 0.05 compared with IL-1β plus IL-6 plus IL-23 plus TGFβ1 control. The comparisons were determined by one-way ANOVA followed by Bonferroni's multiple comparison test **(A,B,D)** or two-tailed unpaired *t*-test **(F)**.

Increased proportions of CD4^+^ T cells expressing IL-17A were present in the peripheral blood from patients with COPD compared with HC and HS (Figure 6A). We further evaluated the concentrations of IL-1β, IL-6, IL-23, and TGFβ1 in HC, HS, COPD patients and found that concentrations of IL-1β, IL-6, and IL-23 in patients with COPD were much higher than those in HC and HS; however, the level of TGFβ1 was significantly decreased in patients with AECOPD (Figure 6B). The above results suggest that these cytokines create a proinflammatory milieu that is thought to be conducive to the generation and differentiation of Th17 cells in COPD patients.

To verify our hypothesis, we utilized a combination of IL-6, IL-1β, IL-23, and TGFβ1 in the absence or presence of inducible CD4^+^CD25^–^Foxp^+^ T cells to induce human Th17 cells *in vitro*. Compared with IL-2-containing medium, the combination of IL-6 plus IL-1β plus IL-23 plus TGFβ1 promoted the differentiation of naive CD4^+^ T cells into Th17 cells; interestingly, culture with CD4^+^CD25^−^Foxp3^+^ T cells resulted in significant expansions of IL-17A^+^ T cells compared with the corresponding combination of different cytokines (Figures 6C,D). Furthermore, inducible CD4^+^CD25^−^Foxp^+^ T cells decreased the expression of CD62L and CCR7 on naïve CD4^+^ T cells, suggesting that CD4^+^CD25^−^Foxp3^+^ T cells-driven proliferation of naïve T cells further caused phenotypic conversion into effector T cells (Figures 6E,F).

**Figure 7:**
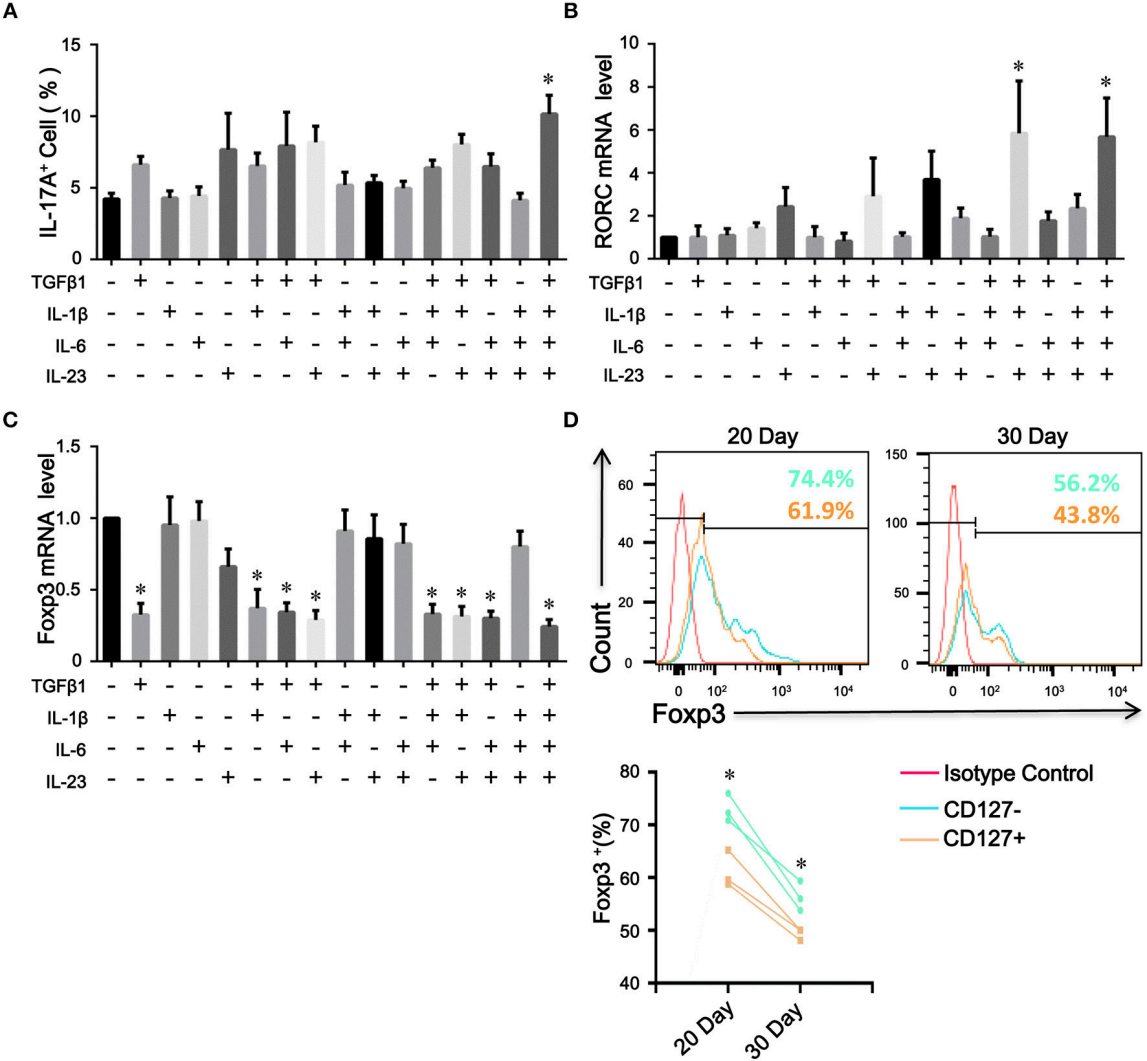
The plasticity and stability of inducible CD4^+^Foxp3^+^ T cells. Inducible CD4^+^CD25^+^ Foxp3^+^ T cells from COPD patients were cultured in the presence of the indicated cytokines, either alone or in various combinations. 7 days after stimulation, the percentages of IL-17A^+^ T cells were analyzed by flow cytometry and Foxp3 and RORC mRNA expression was detected by quantitative real-time PCR. **(A)** Comparisons of percentages of IL-17A^+^ T cells in total cells stimulated by a single cytokine or a combination of various cytokines were performed. Summary data of RORC **(B)** and Foxp3 **(C)** mRNA expression levels were analyzed. The results are reported as means ± SEM from 5 independent experiments, ^*^P < 0.05 compared with medium control. **(D)** Inducible CD4^+^CD25^−^Foxp3^+^ T cells from COPD patients were sorted into CD4^+^CD25^–^CD127^–^Foxp3^+^ T cells (CD127^−^) and CD4^+^CD25^–^CD127^+^Foxp3^+^ T cells (CD127^+^), and Foxp3 stability over time was detected by flow cytometry; ^*^P < 0.05 compared with CD4^+^CD25^–^CD127^+^Foxp3^+^ T cells (CD127^+^) at the same time point. The comparisons were determined by one-way ANOVA followed by Bonferroni‘s multiple comparison test **(A–C)** or two-tailed unpaired *t*-test **(D)**.

Because the levels of proinflammatory cytokines, such as IL-1β, IL-6, and IL-23, were elevated in COPD patients, we assessed the contribution of these cytokines to the plasticity of inducible CD4^+^CD25^+^Foxp3^+^ T cells from COPD patients. As expected, IL-1β, IL-6, or IL-23 combined with TGFβ1 increased the differentiation of IL-17A^+^ T cells from inducible CD4^+^CD25^+^Foxp3^+^ T cells; in contrast, IL-1β plus IL-6 plus IL-23 plus TGFβ1 induced more IL-17A^+^ T cells and higher RORC mRNA expression (Figures 7A,B; Figure S4). Moreover, a fraction of IL-17A^+^ T cells were CD4^+^CD25^–^Foxp3^+^ T cells (Figure S5).

### CD4^+^CD25^−^Foxp3^+^ T Cells Spatially and Temporally Represent an Intermediate Stage of Natural Life-Cycle of T Cells in COPD Patients

**Figure 8:**
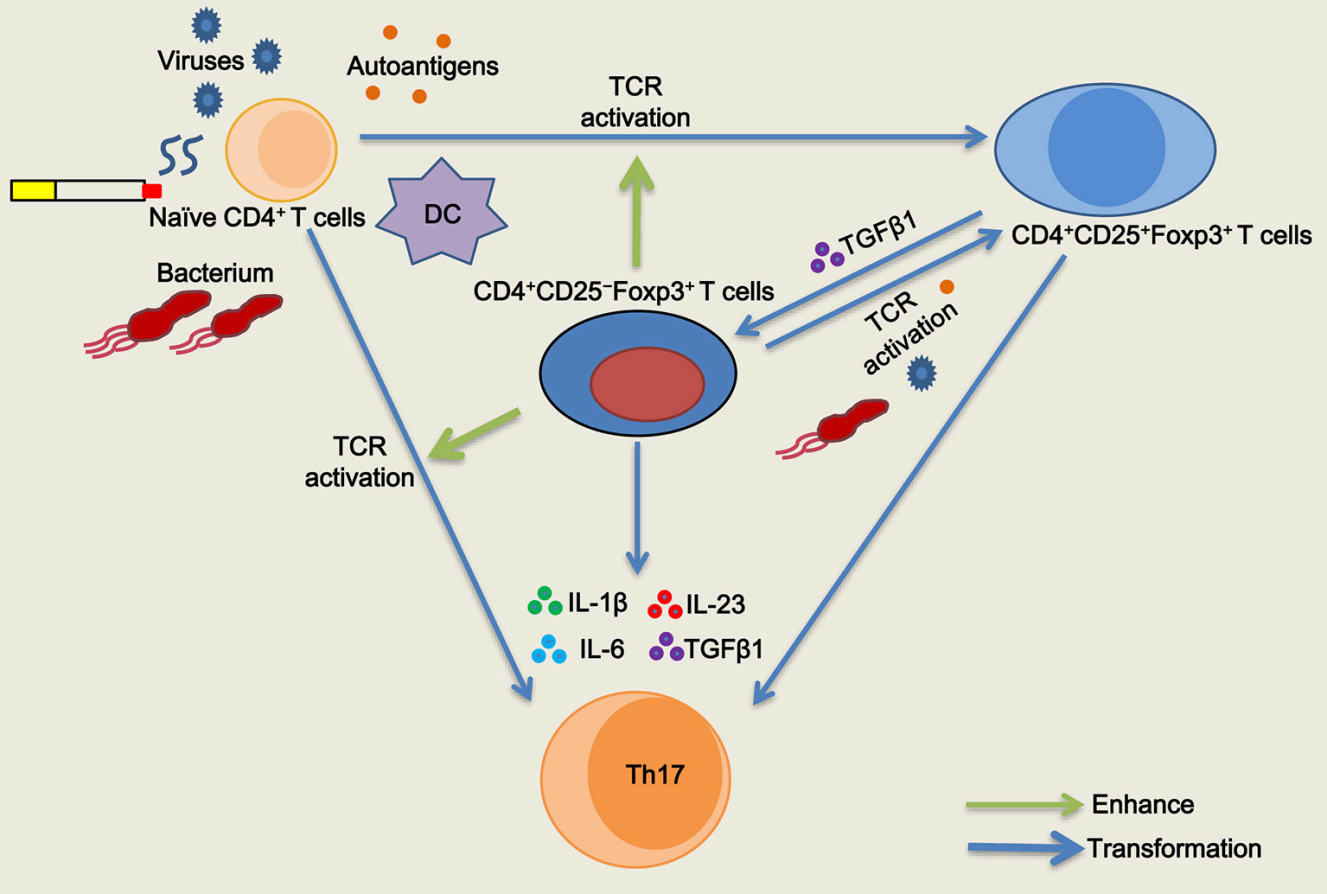
Schematic representation of the role of CD4^+^CD25^−^Foxp3^+^ T cells in T cell homeostasis in chronic obstructive pulmonary disease (COPD). Naïve CD4^+^ T cells are activated and differentiated into CD4^+^CD25^+^Foxp3^+^ T cells when subjected to antigenic stimulation (viruses, bacterium, autoantigens, etc.) in COPD patients, especially during acute exacerbation. After resolution of the exacerbation, TGFβ1 reduces CD25 expression and generates CD4^+^CD25^−^Foxp3^+^ T cells from CD4^+^CD25^+^Foxp3^+^ T cells in stable COPD patients. CD4^+^CD25^−^Foxp3^+^ T cells regain their CD25 expression and functionally enhance the proliferation of naive CD4^+^ T cells by cell contact when T cell receptor (TCR) is simultaneously triggered. CD4^+^CD25^−^Foxp3^+^ T cells facilitate the proliferation and differentiation of naïve CD4^+^ T cells into Th17 cells in the presence of IL-1β plus IL-6 plus IL-23 plus TGFβ1. Meanwhile, a fraction of CD4^+^CD25^−^Foxp3^+^ T cells can be transformed into Th17 cells in the pro-inflammatory cytokine milieu.

The Treg specific demethylated region (TSDR) stabilizes Foxp3 expression (25). Approximately 57.1% of peripheral CD4^+^CD25^−^Foxp3^+^ T cells were reportedly demethylated at the TSDR (18), indicating that a fraction of these cells was unstable *in vivo*. We next sought to determine whether inducible CD4^+^CD25^–^Foxp3^+^ T cells from COPD patients could maintain Foxp3 expression *in vitro*. As with the decreased expression of CD25, we found that TGFβ1 reduced Foxp3 mRNA expression in inducible CD4^+^CD25^+^Foxp3^+^ T cells during the following 7-day cultures; however, IL-1β, IL-6, or IL-23 didn't influence Foxp3 mRNA expression (Figure 7C). Then, we sorted inducible CD4^+^CD25^−^Foxp3^+^ T cells into CD127^+^ and CD127^−^ cells and examined their Foxp3 stability. In contrast to CD4^+^CD25^–^CD127^–^Foxp3^+^ T cells, CD4^+^CD25^−^CD127^+^Foxp3^+^ T cells expressed lower levels of Foxp3 over time (Figure 7D), indicating that both of them could be gradually converted into CD4^+^CD25^–^CD45RO^+^Foxp3^−^ T cells. Additionally, inducible CD4^+^CD25^−^Foxp3^+^ T cells and peripheral CD4^+^CD25^−^CD127^−^CD45RO^+^ T cells rapidly regained their CD25 expression when the TCR was triggered again (Data is not shown). Altogether, these results indicate that circulating CD4^+^CD25^−^Foxp3^+^ T cells at least partially represent an intermediate stage of natural life-cycle of T cells and might perpetuate chronic inflammation in COPD patients (Figure 8).

## Discussion

The enigma always exists in the pathophysiological process of COPD: how inflammation is initiated and why inflammatory response persists. Adaptive immunity plays important roles in the development and progression of COPD. Herein, we first present evidence that CD4^+^CD25^–^Foxp3^+^ T cells differentiate from CD4^+^CD25^+^Foxp3^+^ T cells via the influence of TGFβ1, and then propose a novel mechanism regarding how Th17 cells develop from the transdifferentiation of CD4^+^CD25^−^ Foxp3^+^ T cells in COPD patients. Moreover, the formation of positive feedback loop wherein memory CD4^+^CD25^−^Foxp3^+^ T cells functionally facilitate the differentiation and proliferation of naïve CD4^+^ T cells into Th17 cells might perpetuates chronic inflammation in COPD.

Consistent with the role of CD4^+^CD25^−^Foxp3^+^ T cells in the pathogenesis of SLE (18–20, 22, 23), we expanded this observation to patients with COPD, suggesting a common mechanism of these cells in mediating chronic inflammation. Because CD4^+^CD25^low/−^Foxp3^+^ T cells are derived from CD4^+^CD25^high^Foxp3^+^ T cells in SLE patients, we could obtain the high purity of inducible CD4^+^CD25^+^Foxp3^+^ T cells *in vitro* that helps us study the origin of CD4^+^CD25^–^Foxp3^+^ T cells. Herein, there are some explanations for this phenomenon that a high level of Foxp3 expression was induced when naïve CD4^+^ T cells were activated through TCR crosslinking independent of exogenous TGFβ1. Firstly, reactive oxygen species (ROS) produced by TCR-activated CD4^+^CD25^–^ T cells has a role in producing active TGFβ1, and endogenous TGFβ1 production in turn induces Foxp3 expression in human CD4^+^CD25^−^ T cells (26). Secondly, we can easily obtain the high purity of inducible CD4^+^CD25^+^Foxp3^+^ T cells if T cells are mixed with the pipette 10 times every two days during culture. When naïve T cells are stimulated by anti-CD3/CD28 mAb, not all T cells are equally sensitive to TCR activation. But, the interplay between early activated T cells and naïve T cells can accelerate the activation after T cells are mixed well during culture. Furthermore, Michal Polonsky et al. also observed that local interactions between T cells after TCR stimulation serve as another potent modulator of memory induction *in vivo* and *in vitro* (27). Recent studies have defined TGFβ1 as a critical player in peripheral T cell homeostasis, T cell differentiation and maintenance of Foxp3 expression during the immune response (28). However, our data were not consistent with those reports, which showed that the costimulatory effects of TGFβ1 on naive CD4^+^ T cells upregulated CD25 and cytotoxic T lymphocyte-associated antigen 4 (CTLA-4) expression, increasing the transition of these cells to the activated phenotype (29, 30). Instead, we found that TGFβ1 promptly reduced CD25 expression and generated CD4^+^CD25^−^Foxp3^+^ T cells from CD4^+^CD25^+^Foxp3^+^ T cells *in vitro*.

Comparative phenotypic analysis reveals that both inducible and peripheral CD4^+^CD25^–^Foxp3^+^ T cells globally exhibit the features of activated conventional T cells. The previous studies suggest that human Foxp3^+^ T cells may not be functionally homogenous. Foxp3 is transiently expressed by activated CD4^+^CD25^–^ T cells (9–12), whereas a stable Foxp3 expression is associated with the acquisition of regulatory T cell function (31). Our data is consistent with the previous study that inducible CD4^+^CD25^+^Foxp3^+^ T cells gradually lost CD25 and Foxp3 expressions (9). However, we also observed that some CD4^+^CD25^−^Foxp3^+^ T cells had a stable Foxp3 expression over time, suggesting that these cells were heterogeneous and might contain bona fide inducible Tregs population. Moreover, inducible CD4^+^CD25^+^Foxp3^+^ T cells lost CD25 expression when cultured in serum-free medium in the presence of IL-2, and the effect was delayed by a neutralizing anti-TGFβ1 mAb, suggesting that these cells could secret TGFβ1. Given these facts, we tend to think of CD4^+^CD25^–^Foxp3^+^ T cells as activated conventional T cells. But, we still have reservations that CD4^+^CD25^–^Foxp3^+^ T cells might contain a fraction of bona fide Tregs population or that Tregs are just a special phase in the natural life-cycle of T cells.

Phenotypic analysis suggests that inducible CD4^+^CD25^−^Foxp3^+^ T cells display a phenotype that is consistent with stem cell memory or central memory T cells, whereas peripheral CD4^+^CD25^−^ Foxp3^+^ T cells in COPD patients resemble central memory or effector memory T cells. It is reported that memory T cells cause precocious differentiation of naïve cells through a contact-dependent mechanism involving a non-apoptotic Fas-FasL interaction (32). Our functional assays also indicated that memory CD4^+^CD25^−^Foxp3^+^ T cells enhanced the proliferation of naïve CD4^+^ T cells in cell-dose, contact, and activation dependent manner *in vitro*. We speculated that memory CD4^+^CD25^−^Foxp3^+^ T cells-driven precocious differentiation of naive cells might overcome the immunosuppressive function exerted by TGFβ1. Tu et al. show that active TGFβ signaling is present in naïve T cells, and strong TCR stimulation abolishes TGFβ signaling to overcome its ongoing inhibition, through downregulation of TGFβRI expression (33). Although CD127 expression inversely correlates with the suppressive function of Tregs, our findings show that the enhanced proliferation response conferred by CD4^+^CD25^–^CD127^–^Foxp3^+^ T cells is higher than that by CD4^+^CD25^–^CD127^+^Foxp3^+^ T cells. Regretfully, we currently have no explicit explanation for the functional discrepancy between CD4^+^CD25^–^CD127^+^Foxp3^+^ T cells and CD4^+^CD25^−^CD127^−^Foxp3^+^ T cells.

To date, there are no studies examining the possible role of CD4^+^CD25^−^Foxp3^+^ T cells in the pathogenesis of COPD. IL-17A^+^ T cells induce a severe neutrophilic response in COPD (34, 35). Our data are consistent with those previously reported by others, showing that a higher proportion of CD4^+^ T cells expressing IL-17A is observed in the peripheral blood from COPD patients and correlates with a proinflammatory cytokine milieu (4, 5). Importantly, we found that CD4^+^CD25^−^Foxp3^+^ T cells can facilitate the differentiation and proliferation of naïve CD4^+^ T cells into Th17 cells in this microenvironment. Memory T cell-driven differentiation of naive cells might perpetuate chronic inflammation seen in patients with COPD. Hence, frequent exacerbations, whether caused by bacterium or viruses (36), contribute to the activation of T cells that helps to amplify the interactions between naive CD4^+^ T cells, CD4^+^CD25^−^Foxp3^+^ T cells, Th17 and neutrophil in COPD patients. The hallmark of COPD is also the development of exaggerated chronic inflammation in the lung in response to inhalation of cigarette smoke (37). Cigarette smoke extract-induced neutrophil extracellular traps are found to drive plasmacytoid dendritic cell maturation and activation, initiating a T-cell-mediated immune response (38). We observed that the number of peripheral CD4^+^CD25^−^Foxp3^+^ T cells in smokers without lung disease was much higher than in HC subjects, suggesting that smokers without clinical signs have an increased susceptibility to develop COPD with the continued accumulation of inflammation; it is possible that these cells arose due to certain autoantigen stimulation. Therefore, avoiding frequent exacerbations and smoking cessation are effective in postponing the decline of lung function and decreasing mortality. Taken together, our results suggest that the formation of a proinflammatory microenvironment might be the main culprit, resulting in a vicious cycle in which many CD4^+^CD25^−^Foxp3^+^ T cells induced by cigarette smoke or other factors in turn facilitated the differentiation and proliferation of naïve CD4^+^ T cells into Th17 cells, which eventually contributes to the amplification and perpetuation of chronic inflammation in COPD.

Massive evidence suggests that CD4^+^ T cells, particularly iTreg and Th17 cells, are more plastic than originally appreciated (39, 40). We found that inducible CD4^+^CD25^+^Foxp3^+^ T cells lost CD25 and Foxp3 expression and readily acquired RORC expression when subjected to Th17 cell-polarizing conditions. The decreased expression of Foxp3 is likely caused by less responsiveness to IL-2 due to the down-regulation of CD25. Down-regulation of Foxp3 depends on the ubiquitination by the E3 ligase Stub1 (41), and Foxp3 instability leads to the generation of pathogenic memory T cells (42). Cytokine-driven proliferation of stem cell memory and central memory T cells causes phenotypic conversion into effector memory T cells (43). CD4^+^CD25^−^Foxp3^+^ T cells comprise uncommitted Tregs that can be instructed by cytokines to differentiate into effector T cells (44). These findings further verify that the increased proportion of Th17 cells in COPD patients might be due not only to the differentiation of naïve CD4^+^ T cells but also to the conversion of CD4^+^CD25^−^ Foxp3^+^ T cells in the proinflammatory cytokine milieu.

However, many limitations need to be taken into consideration in our study. Firstly, most CD4^+^CD25^+^Foxp3^+^ T cells, which express CD45RO, transdifferentiate into CD4^+^CD25^−^Foxp3^+^ T cells via TGFβ1 stimulation; whether TGFβ1 can influence CD25 expression on CD4^+^CD25^+^CD45RA^+^Foxp3^+^ T cells remains obscure. Secondly, the molecular mechanism about how TGFβ1 regulates CD25 expression or accelerate proteolytic cleavage remains to be investigated. Moreover, CD4^+^CD25^−^CD45RO^+^CD127^+^ T cells and CD4^+^CD25^−^CD45RO^+^CD127^−^ T cells from peripheral blood were used as substitutes for CD4^+^CD25^−^CD127^+^Foxp3^+^ T cells and CD4^+^CD25^−^CD127^−^Foxp3^+^ T cells for functional assays; these cells couldn‘t represent bona fide CD4^+^CD25^−^Foxp3^+^ T cells due to their low expression of Foxp3. Lastly, the main limitation of our study is that the *in vitro* demonstrated mechanisms/consequences of these cells are from the small number of COPD patients; it needs further study to validate whether these results can be widely available to all COPD patients.

In conclusion, our study demonstrates, for the first time, that TGFβ1 decreases CD25 expression and is responsible for the generation of CD4^+^CD25^−^Foxp3^+^ T cells from CD4^+^CD25^+^ Foxp3^+^ T cells. Functional assays indicate that memory CD4^+^CD25^−^Foxp3^+^ T cells facilitate the differentiation and proliferation of naïve CD4^+^ T cells into effector Th17 cells in a proinflammatory cytokine milieu. Furthermore, several CD4^+^CD25^−^Foxp3^+^ T cells retain the ability to differentiate into pathogenic Th17 cells and might perpetuate chronic inflammation in COPD. Finally, our findings suggest that CD4^+^CD25^−^Foxp3^+^ T cells might provide a potential biomarker for assessing disease activity in COPD patients. Hence, a large-scale prospective cohort study needs to be designed to validate CD4^+^CD25^−^Foxp3^+^ T cells as biomarkers of disease activity in COPD patients followed up longitudinally.

## Author Contributions

X-ZX designed the study and experiments. J-HW, MZ, Z-JM, S-WS, S-YM, and H-LH were responsible for sample collection, cell isolation and culture, and flow cytometry. J-HW, X-ZX, YJ, and X-NT analyzed the data. J-HW and X-ZX drafted the manuscript.

### Conflict of Interest Statement

The authors declare that the research was conducted in the absence of any commercial or financial relationships that could be construed as a potential conflict of interest.
