# Supplementary material for: Generation and Immune Regulation of CD4+CD25−Foxp3+ T Cells in Chronic Obstructive Pulmonary Disease
Source: Front Immunol. 2019 Feb 20;10:220. doi: 10.3389/fimmu.2019.00220 (PMC6392103; doi:10.3389/fimmu.2019.00220)
Supplement: Supplementary file 1 [file Table_1.DOCX]

**Supplementary figure legends**

**Figure S1.** Gating strategies. Related to Figure 3A. **(A)** Gating strategy for CD4**+** T cell subsets based on CD25 and Foxp3 expression. **(B)** Representative dot plots for the detection of the distribution of CD45RA, CD45RO, CD62L, CD69, CD95, CD127 and PD-1 amongst: peripheral CD4+ T cells subsets (CD4+CD25−Foxp3− T cells, CD4+CD25+Foxp3− T cells, CD4+CD25+Foxp3+ T cells and CD4+CD25−Foxp3+ T cells) from SCOPD patients (n=8) and inducible CD4+CD25−Foxp3+ T cells (Inducible, n=5).

**Figure S2.** Analysis and FACS isolation of CD4+CD25−CD127+CD45RO− T cells, CD4+ CD25−CD127+CD45RO+ T cells and CD4+CD25−CD127−CD45RO+ T cells. **(A)** CD4+ CD25−CD127−CD45RO+ T cells (S1), CD4+CD25−CD127+CD45RO+ T cells (S2) and CD4+ CD25−CD127+CD45RO− T cells (S3) were isolated by FACS. Representative dot plots (pre-sort and post-sort) are shown. **(B)** The comparison of Foxp3 expression levels in CD4+ CD25−CD127−CD45RO+ T cells (S1) and CD4+CD25−CD127+CD45RO+ T cells (S2) from peripheral blood in COPD patients were performed. The results represent the means ± SD from 10 independent experiments. The comparisons were made using two-tailed unpaired *t*-test. A value of *P* < 0.05 was considered statistically significant.

**Figure S3.** CD4+CD25−CD45RO+CD127+ T cells and CD4+CD25−CD45RO+CD127− T cells from COPD patients exhibit regulatory activity equal to those cells from HC subjects. Representative histograms showing the proliferation of CFSE-labeled naïve CD4+ T cells from COPD patients **(A)** or HC **(B)**, activated with plate-bound anti-CD3/CD28 Abs, were co-cultured with the indicated conditions of unlabeled CD4+CD25−CD127−CD45RO+ T cells (CD127−) and CD4+CD25−CD127+CD45RO+ T cells (CD127+) from COPD patients or HC (1:1 ratio). 4 days after stimulation, the proliferation was evaluated by flow cytometry. Graphs **(C)** and **(D)** represented summary data **(A)** and **(B)**, respectively. Graphs showing the percentages of the enhancement of proliferation by peripheral CD4+CD25−CD4RO+CD127+ T cells (CD127+) and CD4+CD25−CD45RO+CD127− T cells (CD127−) in each group, indicated by progressive numbers. The results are reported as means ± SEM from 3 independent experiments. The comparison was made using two-tailed unpaired *t*-test; a value of *P* < 0.05 was considered statistically significant.

**Figure S4.** Gating strategies. Related to Figure 6A. Inducible CD4+CD25+Foxp3+ T cells from COPD patients were cultured in the presence of the indicated cytokines, either alone or in various combinations. 7 days after stimulation, the percentages of IL-17A+ T cells were analyzed by flow cytometry. **(A)** Representative dot plots gated on T cells are from one of 5 independent experiments.

**Figure S5.** A fraction of IL-17A+ T cells are CD4+CD25−Foxp3+ T cells. Inducible CD4+ CD25+Foxp3+ T cells were cultured in the presence of IL-1β plus IL-6 plus IL-23 plus TGFβ1. 7 days after culture, the cells were stimulated with PMA and ionomycin for 5 h and analyzed for CD25, IL-17A and Foxp3 expression. **(A)** Th17 cells were identified based on their expression of IL-17A; **(B)** A representative dot plot from one of 3 independent experiments showing that a fraction of IL-17A+ cells were CD4+CD25−Foxp3+ T cells.
