# Supplementary material for: Generation and Immune Regulation of CD4+CD25−Foxp3+ T Cells in Chronic Obstructive Pulmonary Disease
Source: Front Immunol. 2019 Feb 20;10:220. doi: 10.3389/fimmu.2019.00220 (PMC6392103; doi:10.3389/fimmu.2019.00220)
Supplement: Supplementary file 2 [file Table_2.docx]

**Table 1. List of antibodies**

| Antibodies | Source | Clone | Identifier |  |
| --- | --- | --- | --- | --- |
| anti-CD3-PerCP-Cy5.5  anti-CD4-FITC  anti-CD8-FITC  anti-CD25-PE-Cy7  anti-CD45RA-APC  anti-CD45RA-BV421  anti-CD45RO-PerCP-Cy5.5  anti-CD45RO-BV421  anti-CD62L-BV421  anti-CD69-BV421  anti-CD95-BV421  anti-CD127-BV421  anti-CD127-PE  anti-PD-1-BV421  anti-CCR7-PE-Cy7  anti-Helios-APC  anti-TIGIT-APC  anti-CTLA-4-BV421  anti-KI-67-APC  anti-Foxp3-PE  anti-Foxp3-APC  anti-IL-17A-APC  Fixable viability stain 450 | BD  BD  BD  BD  BD  BD  BD  BD  BD  BD  BD  BD  BD  BD  BD  eBioscience  eBioscience  BD  eBioscience  eBioscience  eBioscience  eBioscience  BD | UCHT1  RPA-T4  HIT8a  M-A251  HI100  HI100  UCHL1  UCHL1  DREG-56  FN50  DX2  HIL-7R-M21  HIL-7R-M21  EH12.1  3D12  22F6  MBSA43  BNI3  20Raj1  236A/E7  236A/E7  eBio64DEC17 | 560835  555346  555634  557741  561884  562885  560607  562641  563203  562884  562616  562437  562436  562516  560922  17-9883-41  17-9500-41  562743  17-5699-41  12-4776-41  12-4777-41  17-7179  562247 |  |

**Table 2. List of primers**

| Gene name | Primer sequence (5`-3`) |
| --- | --- |
| Foxp3 | F: CTGGCAAATGGTGTCTGCAAGT |
|  | R: CTGCCCTTCTCATCCAGAAGATG |
| RORC | F: CTGCAAGACTCATCGCCAAAG |
|  | R: TTTCCACATGCTGGCTACACA |
| CD25 | F: TTCATACCTGCTGATGTGGGG |
|  | R: TTGGTTGTCCCAGGACGAGT |
| GAPDH | F: GCACCGTCAAGGCTGAGAAC |
|  | R: TGGTGAAGACGCCAGTGGA |
