# Supplementary figures and images for: Generation and Immune Regulation of CD4+CD25−Foxp3+ T Cells in Chronic Obstructive Pulmonary Disease
Source: Front Immunol. 2019 Feb 20;10:220. doi: 10.3389/fimmu.2019.00220 (PMC6392103; doi:10.3389/fimmu.2019.00220)

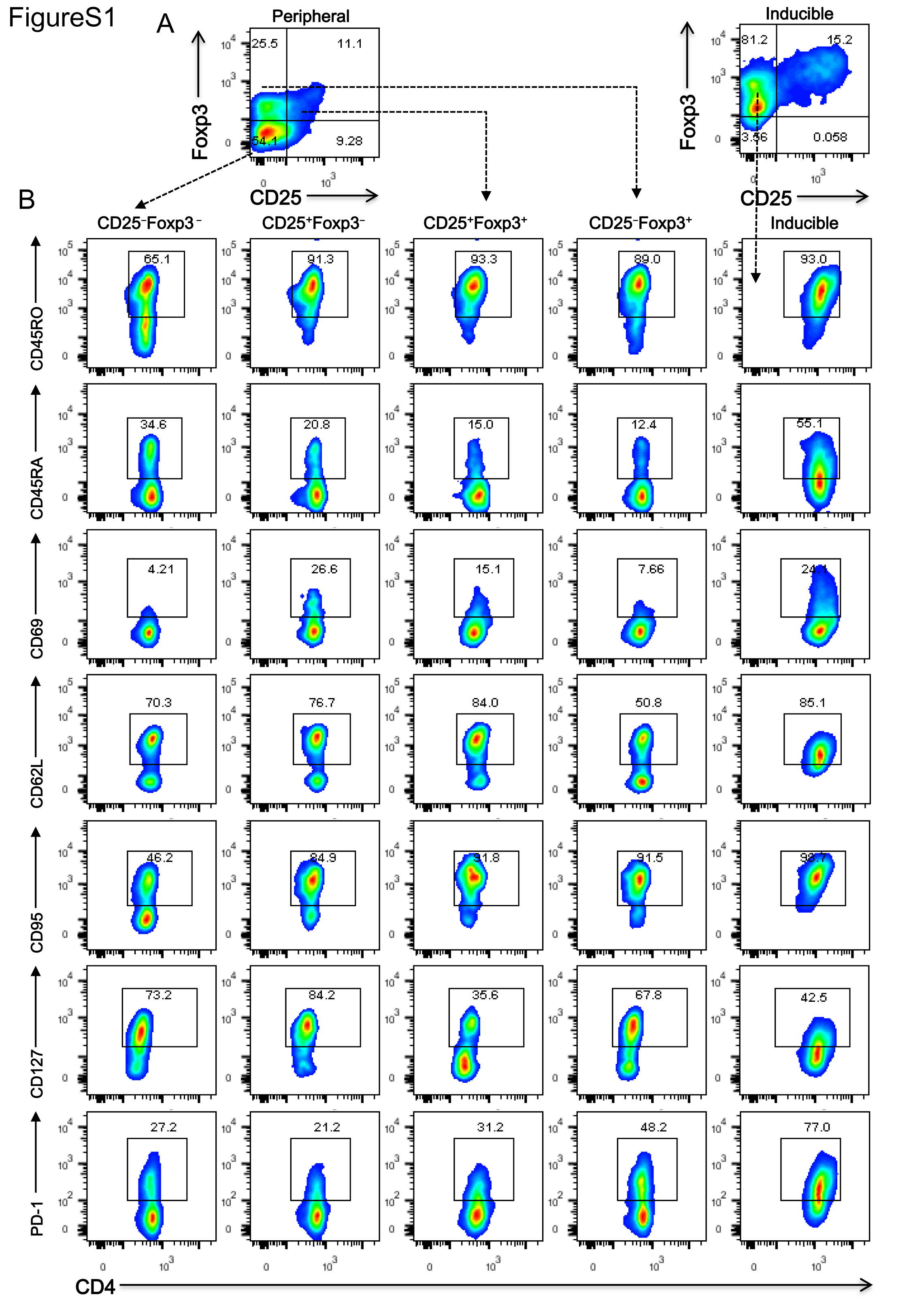

Supplement: Supplementary file 3 [file Image_1.TIF]

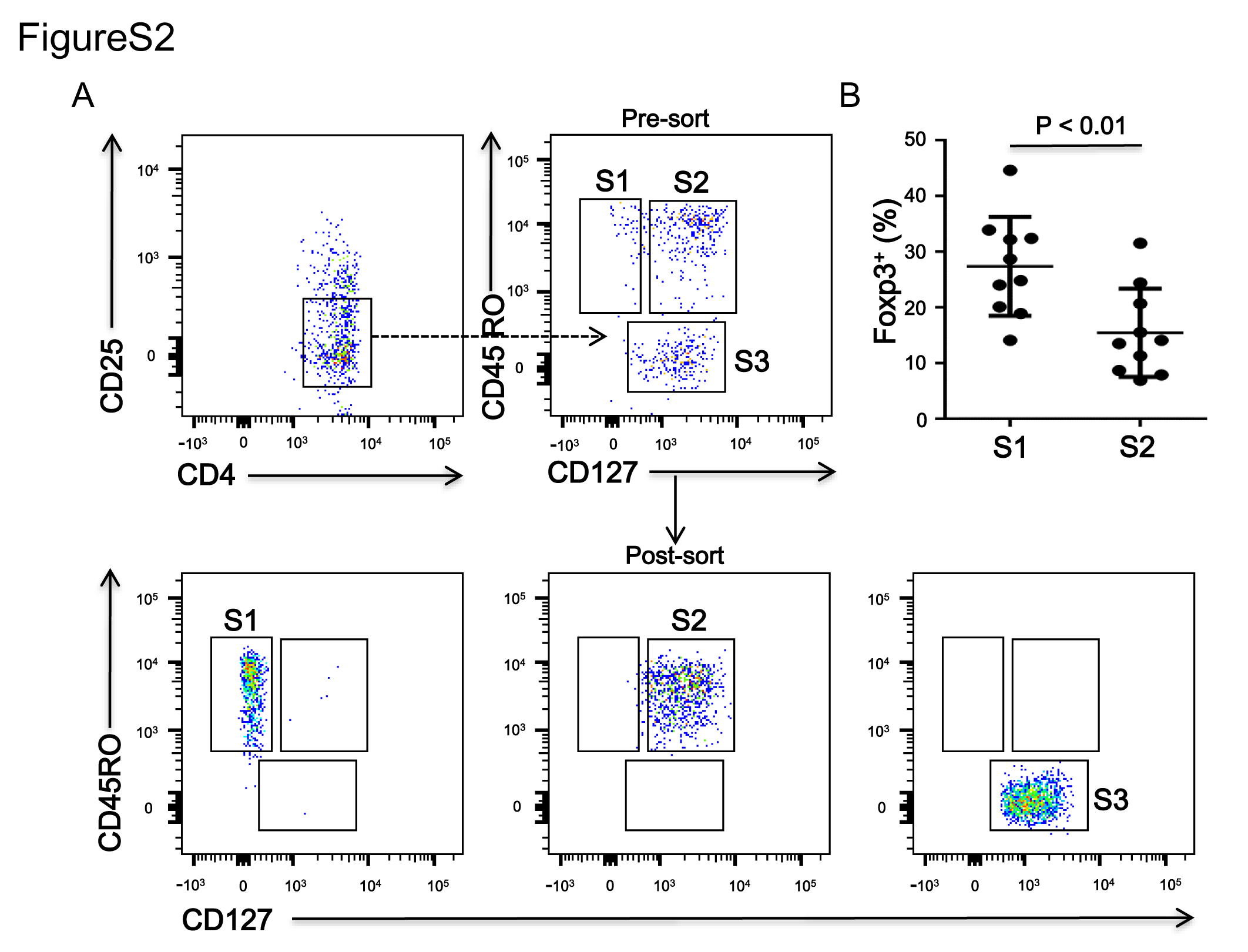

Supplement: Supplementary file 4 [file Image_2.TIF]

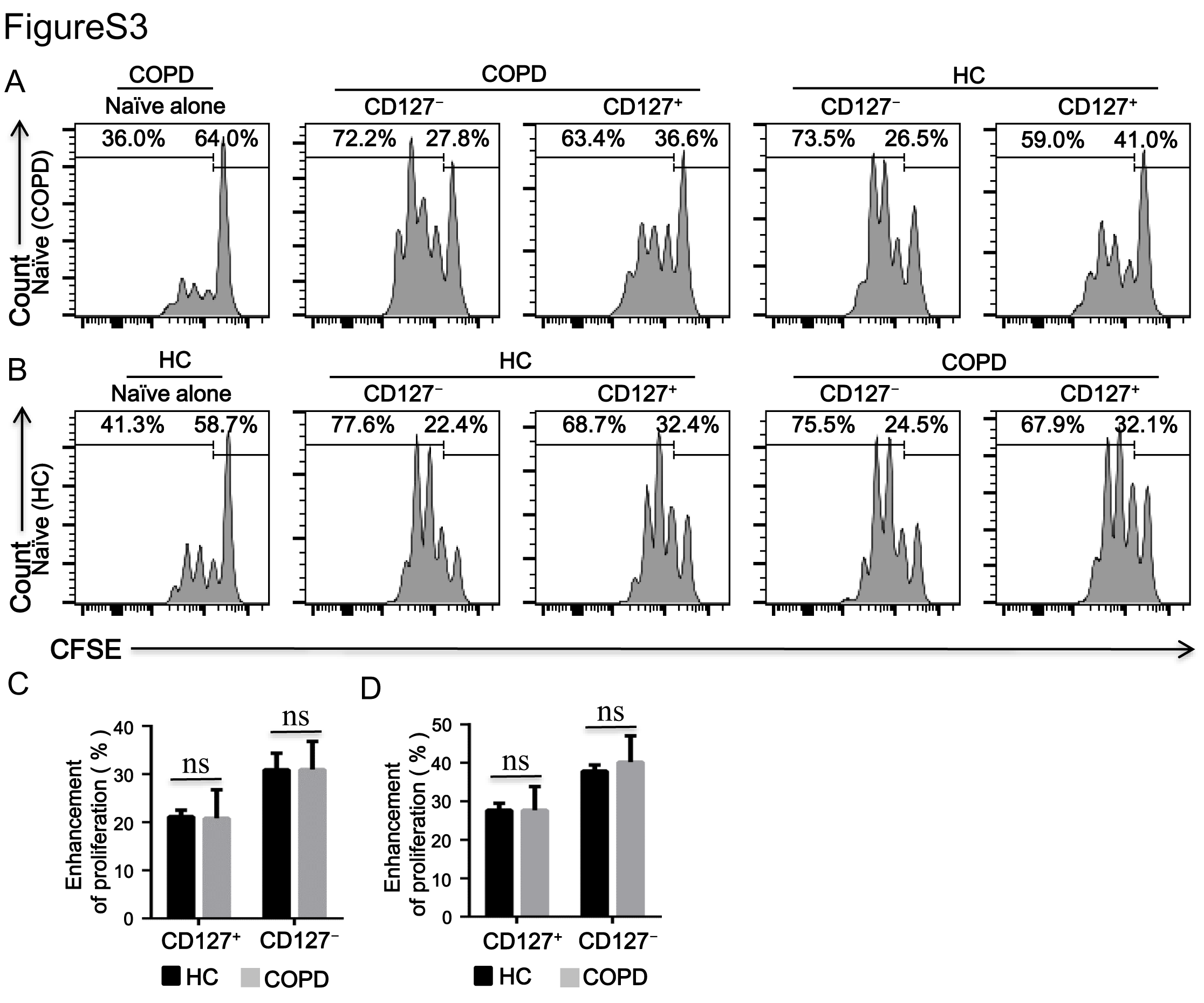

Supplement: Supplementary file 5 [file Image_3.TIF]

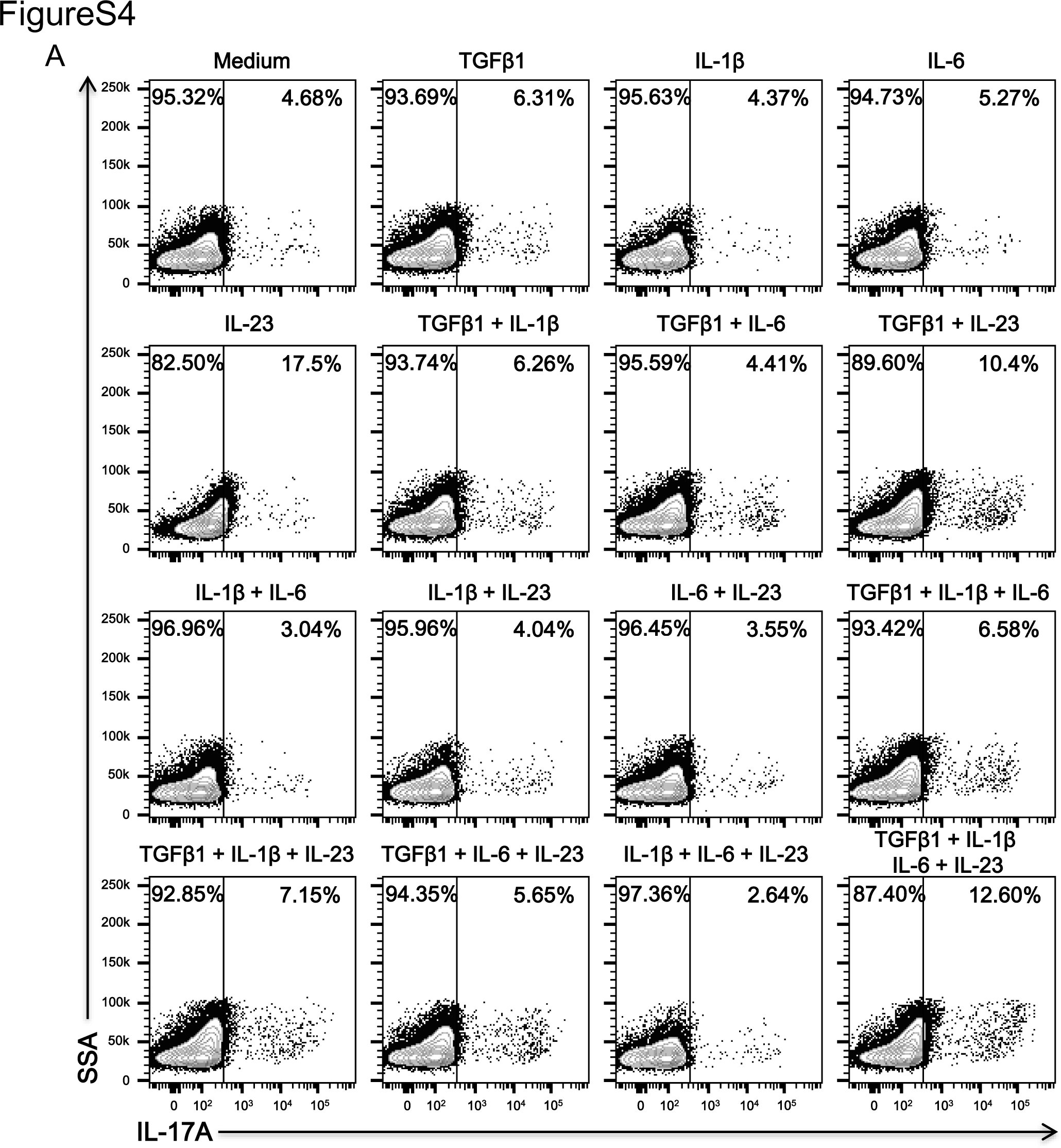

Supplement: Supplementary file 6 [file Image_4.TIF]

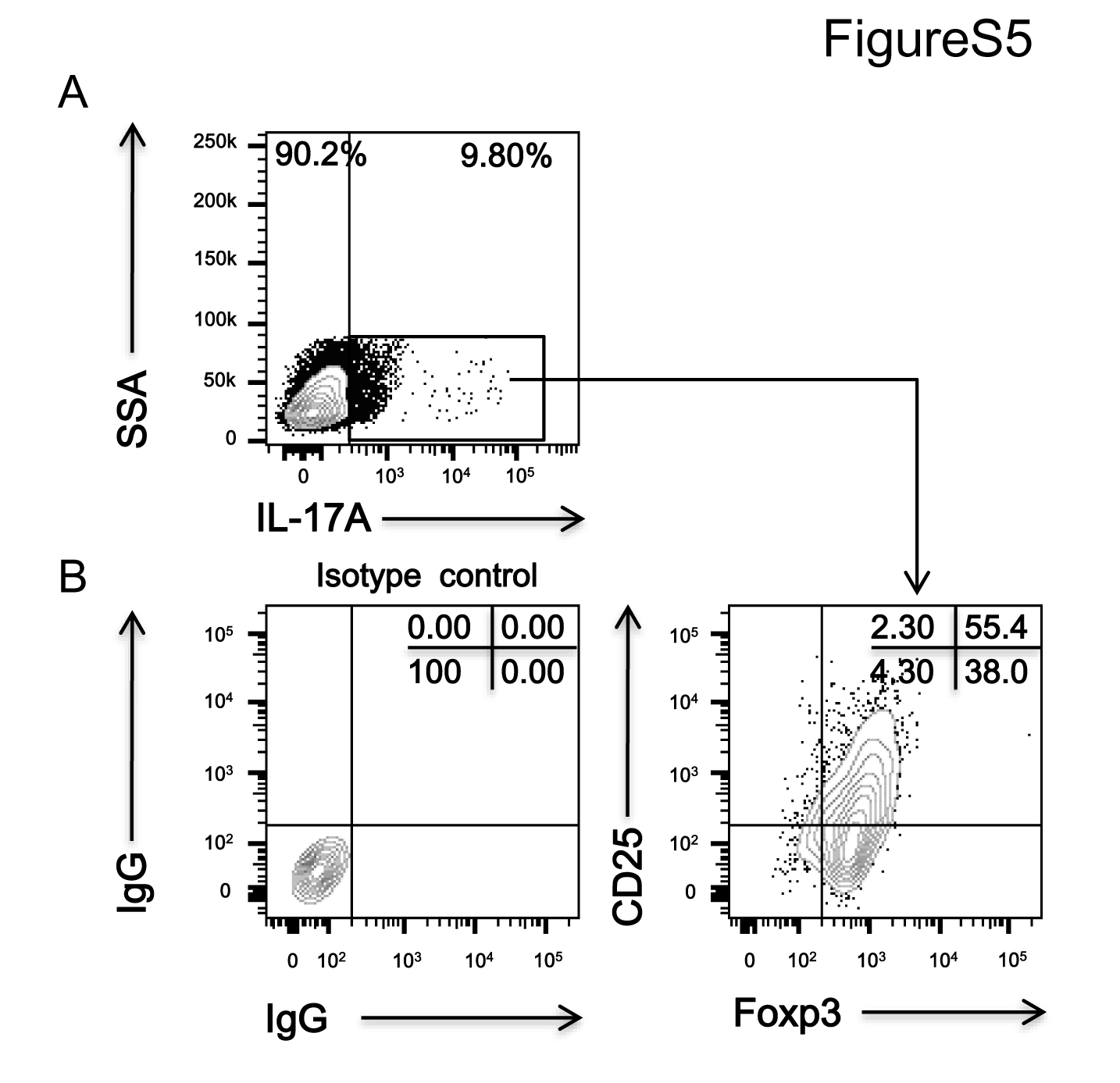

Supplement: Supplementary file 7 [file Image_5.TIF]
